# Ingroup love, outgroup hate, and the gateway group effect: Comparing the direct and indirect impact of dual versus single identification

**DOI:** 10.1371/journal.pone.0287631

**Published:** 2023-08-16

**Authors:** Aharon Levy, Adam Galinsky, Christine Q. Nguyen, Tamar Saguy, Elif G. Ikizer, John F. Dovidio

**Affiliations:** 1 Columbia Business School, Columbia University, New York, New York, United States of America; 2 Department of Psychology, Yale University, New Haven, Connecticut, United States of America; 3 Department of Psychology, Reichman University, Herzliya, Israel; 4 Department of Psychology, University of Wisconsin, Green Bay, Wisconsin, United States of America; Goethe University Frankfurt am Main, GERMANY

## Abstract

Decades of research in social identity have shown that people instinctively hold positive attitudes towards ingroup members and negative attitudes towards outgroup members. However, it remains unclear how people respond to individuals explicitly identified with both one’s ingroup and outgroup. We propose that when people are exposed to dual-identified individuals and groups (e.g., Muslim-Americans explicitly identifying with both their Muslim and American identities), intergroup attitudes will improve, driven more by the ingroup component (American), despite the presence of the outgroup component (Muslim). Moreover, we suggest exposure to dual-identification can also improve attitudes toward the broader outgroup (Muslims more generally), a phenomenon called the gateway-group effect. To test these hypotheses, we created a new measure of dual-identification and conducted three studies involving both Muslim-Americans and Mexican-Americans. Results confirmed that exposure to explicitly dual-identified groups improved attitudes towards the dual-identified group (e.g., Mexican-Americans) as well as toward the respective outgroup (e.g., Mexicans).

## Introduction

When France won the 2018 soccer World Cup for the second time in its history, many commentators worldwide noted that more than half of the French team was of African descent. Not surprisingly, African people all over the world celebrated the Africanness of the players, who were of both French citizenship and African heritage. This dual identity even prompted some to suggest that it was, in fact, Africa’s first World Cup victory [1]. By contrast, French officials stated that France does not refer to its citizens based on their origin, race, or religion, and that there is no hyphenated French identity (e.g., African-French players). Instead, they declared that all players were simply French. While this example represents a case of singular accomplishment, it reveals how individuals who possess a dual identity may be perceived and responded to in ways that can have important consequences for intergroup relations generally.

This example highlights an increasingly prevalent phenomenon of dual identification, in which people share group membership with others who hold multiple social identities simultaneously (e.g., people with dual citizenship). The massive growth in social and global interconnections [2] has stimulated a dramatic rise in the presence of individuals and groups with dual or multiple social identities [3; 4]. For instance, in its past three presidencies, the United States has seen a President and Vice President with parents born in different countries and a first lady with dual citizenship. As a result, people are becoming increasingly exposed to individuals holding dual identities, as well as to the notion of dual identity more generally [5].

In the World Cup example, the players with the dual identities were treated by their respective counterparts as ingroup members (i.e., as African by people from Africa and as French by the French) despite the outgroup component of their identity. One may argue that this focus on only the ingroup component of the dual identity was driven by basking in the reflected glory of the World Cup victory [6], which enhanced the status of one’s own group [7]. However, we suggest that this example is not exceptional and represents a larger effect of dual identification.

In the current research, we explore what happens to intergroup attitudes when people are exposed to others who are explicitly identified with two different social groups (one ingroup and one outgroup), such as a French citizen being exposed to African immigrants who explicitly identify with both their French and African identities. We propose that exposure to others who explicitly hold a dual identity will lead intergroup perceptions to be similar to that of the ingroup component despite the outgroup affiliation. In this example, we propose that African immigrants who explicitly identify with both their African and French identities will be treated similarly positively by the French people as immigrants who only identify with being French. Moreover, we further suggest that as a result of exposure to the dual identification, people will also become more positive toward the outgroup associated with that dual identity; in this example, exposure to dual-identified African immigrants in France will lead people from France to have more positive attitudes towards Africans with no French affiliation. This latter effect has been called the gateway group effect because the African component of the African-French dual identity serves as a gateway to improve attitudes towards Africans more broadly [8].

To explore the intergroup consequences of being exposed to dual identification, we conducted three studies using correlation and experimental designs across two intergroup contexts. Prior work has demonstrated the gateway group effect in terms of the identity of others–that is, as a member of one group (e.g., Muslim) or another (e.g., American) or as a member of a group representing a dual identity (e.g., Muslim American)–with minimal groups and without attempting to manipulate perceived identification [8, Studies 2 and 3]. This earlier work examined exposure to a dual identity group (versus not). The current work builds on these findings by holding constant exposure to a dual identity group and examining the effect of perceived identification of the group with the ingroup and outgroup. More specifically, we measure and manipulate the degree to which individuals are seen as identifying with each of the groups represented in their dual identity. This empirical approach allows us to flesh out the driving role of perceived identification in the gateway group effect.

The current research offers a number of novel and substantial contributions to the field, extending work on how people respond to others whom they perceive as having a dual (vs. single) identity by testing the impact of the strength of identification that individuals express for the separate components of their dual identity. First, despite this dramatic rise in exposure to social identities that are explicitly dual in nature, it is not clear how people react to individuals who explicitly identify with both one’s own ingroup and an outgroup (compared to their responses to someone identified with only one of these identities). We address this question by comparing different levels of perceived identification and examine the relative effect of the ingroup and outgroup identity components of the dual identity on intergroup attitudes. In doing so, this research considers whether people with dual social identities can fully embrace both of their identities without necessarily suffering negative effects from their identification with an outgroup. Furthermore, the current research expands the understanding of the gateway group effect by fleshing out the role of perceived identification in driving this effect. Taken together, the current research provides a new theoretical lens to better understand, both conceptually and practically, one of the fastest growing demographic trends today.

## The single identity focus of existing research and practice

People and groups holding a dual social identity are often categorized by others as possessing only one of their identities [9; 10]. The consequence of perceived single identification is that the dual-identified person or group is typically classified by observers as either an ingroup or an outgroup member, even though such individuals may identify with both groups. For example, cross-categorization research has found that when an individual has identities that cut across different dimensions (e.g., gender and race), one dimension tends to be the primary focus in the minds of perceivers [11].

This simplification process is present not only in the minds of research participants but also in the theoretical lens used by researchers. The social identity literature, for instance, has explored the construct of social identification in depth [12]. However, research in this area has either focused on attitudes toward ingroup members as a function how much research subjects are identified with the ingroup [13], or on attitudes toward outgroup members as a function of how much subjects are identified with the outgroup [14]. Rarely has research tested the degree of perceived identification with both the ingroup and the outgroup. This limited attention to dual identification is true even in studies that examine reaction to the identities of minority groups that inherently have dual identities, such as immigrants or biracial individuals.

Although the existing literature informs an understanding of how people are treated when they are perceived as ingroup members versus outgroup members, it offers relatively little insight about what happens when an individual is perceived to be identified with both the ingroup and an outgroup simultaneously. In the context of the African-French World Cup example, little research clarifies the consequences of the players being perceived as identified with both their French and African heritage.

The present research moves the literature forward by examining attitudes of participants (e.g., Americans) towards groups (e.g., Muslim Americans) who are explicitly identified with both the ingroup (Americans) and the outgroup (Muslims). We also examine the attitudes toward the outgroup associated with the dual identity (e.g., non-American Muslims) after exposure to dual-identified groups. As we elaborate next, we propose that, because of the primary importance of the recognition of ingroup membership [11], the effects of a dual identity on intergroup dynamics may often be similar to those of an ingroup identity despite the existence of the outgroup identity. We also propose that exposure to a dual identity group will improve attitudes toward the outgroup that is associated with the dual identity, a phenomenon known as the gateway group effect.

In the present work, we focus on the attitudes of majority-group members toward minority groups, which does raise questions around whether these effects would generalize to the attitudes of minority-group members. While we acknowledge that the attitudes of both majority and minority group members shape dynamics of intergroup relations, the motivation behind the decision to focus on the attitudes of the majority group perspective was the tendency for majority group members to possess the requisite resources and sociopolitical power to create significant social change.

## The intergroup consequences of dual identities

The impact of social identity and categorization has been at the center of intergroup psychology research for over half a century [7; 15; 16]. Thousands of studies have found robust evidence that the categorization of others as part of an ingroup improves attitudes toward those individuals but creates discriminatory attitudes toward those identified with one’s outgroup [12; 17; 18]. However, despite the robustness of these findings, the consequences of perceiving others with a dual identity, specifically in terms of identifying with a perceiver’s ingroup and an outgroup, remain unclear. Theoretically, in cases in which people are exposed to someone explicitly identifying with both one’s ingroup and an outgroup, there can be three possible intergroup attitudes: (a) driven equally and additively by both the ingroup and the outgroup identities, (b) driven mainly by the outgroup identity, or (c) driven mainly by the shared ingroup identity.

The first potential intergroup effect of perceiving an individual or group as explicitly holding a dual identity is additive, driven by both the outgroup and the ingroup identity. This additive possibility predicts, at least for identities equivalent in strength, a neutral impact on intergroup attitudes and behavior. Cross-categorization research has found that having a “crossed” identity of two identities from different dimensions—sharing some social identities (e.g., gender) but not others (e.g., ethnicity)—produces more positive attitudes than having a double outgroup identity (e.g., not sharing either gender or ethnicity), but produces worse attitudes than having a double ingroup identity (e.g., sharing both gender and ethnicity, [19; 20]). For example, cross-categorization research would predict that, compared to an American man, an American woman would evaluate a Mexican woman more positively (because of the shared gender dimension) but would evaluate this person less positively than she would evaluate another American woman (with whom she shares both gender and ethnic dimensions; [21; 22]). If the same processes associated with cross-categorization occur in the context of perceiving dual identities, then the positive component of the ingroup identity and the negative component of the outgroup identity would cancel each other out. That is, dual-identified groups would be perceived less positively than someone identified only with the ingroup but not as negatively as someone identified only with the outgroup.

Importantly, the dual identity framework differs from the cross-categorization framework. Cross-categorization research has traditionally focused on identities that exist across separate dimensions (e.g., gender and ethnicity). However, dual identities often exist along the same dimension and result in complex, hyphenated identities (e.g., nationality for immigrants, such as Mexican-American individuals).

There is also research that goes against the additive prediction and suggests that only one of the perceived identities will inform intergroup attitudes. Research finds that people have more difficulty processing information relating to another person’s dual identity than about an individual’s outgroup or ingroup identity alone [23]. One likely consequence is that people and groups holding dual identities are often perceived by others in terms of only one of their identities, especially when the dual identification is not explicitly stated [24; 25]. With respect to dual identities involving both an ingroup and an outgroup identity, this simplification could lead observers to respond to the individual with a dual identity primarily as an outgroup member or an ingroup member.

Accordingly, another potential intergroup outcome of dual identity is that intergroup perceptions will be driven mainly by the outgroup identity. There are a number of reasons that suggest that this simplification process would lead attitudes toward people with a dual identity to be based primarily on their outgroup identity. In general, people tend to evaluate outgroup members less favorably than ingroup members [26; 27] and tend to perceive outgroup members as competitive [28], manipulative [29], and threatening [30]. In the context of perception and decision making, negative information attracts more attention than positive information and is typically weighted more heavily [31]. With respect to social perception, people strongly weigh negative factors when forming evaluations and impressions of other individuals and groups [32], and people and groups exhibiting combinations of negative and positive qualities are evaluated in ways that are more negative than the algebraic sum would predict [33; 34]. In addition, in intergroup contexts, people attend more strongly to outgroup members than to ingroup members because of the potential costs to the ingroup of misidentification. Furthermore, they tend to exclude individuals from the ingroup when their identities are ambiguous (the ingroup over exclusion effect; [35]). An example of this can be seen when people’s dual identity is based in being mixed race. Research has found that when faced with White and Black biracial people, White observers tend to respond to these individuals based primarily on their racial identity, that is, the outgroup; this perception has been called hypodescent [9; 36; 37]. Indeed, people have been found to react mostly positively to dual-identified individuals who express identification solely with a superordinate group, and react equally negatively to dual-identified individuals who identify solely with the outgroup or who express strong dual identification [38].

In summary, the additive perspective suggests that the impact of dual identification for intergroup relations is driven by both the ingroup and outgroup identity, resulting in zero effect on attitudes towards the dual-identified group; another perspective predicts that the impact will be driven mainly by the outgroup identity, resulting in a negative effect on intergroup attitudes. In contrast, we offer a novel perspective on the impact of dual identification for intergroup relations. We hypothesize that perceptions of gateway individuals and groups that are strongly identified with both an observer’s ingroup and an outgroup would be similar to perceptions of gateway individuals and groups primarily identified an observer’s ingroup.

Our hypothesis draws on work showing that intergroup bias and discrimination are often motivated by ingroup love—preferential treatment of ingroup members—rather than direct hostility toward outgroup members. Although negativity may be more heavily weighted in interpersonal relations, when it comes to intergroup relations, ingroup love trumps outgroup hate [39–41]. Accordingly, negative attitudes toward the outgroup result from a lack of a shared ingroup, whereas a dual-identified individual or group provides a connection to the ingroup. Therefore, we predict that dual-identified individuals and groups will receive the positive attitudinal benefits of sharing the ingroup identity without suffering the full consequences of that person’s identification with the outgroup. Recent research offers some suggestive evidence that supports our prediction. A recent study found that although dual-identified individuals were punished when they were seen as only loyal to the outgroup, in the absence of this disloyalty information, there were no differences in perceptions towards an ingroup member and a dual-identity individual [42, Study 1].

### The gateway group effect

In addition to investigating how people respond to others who possess a dual identity, the current research also examined the impact on intergroup relations more broadly. Research has begun to examine the impact of dual-identified groups as a potential bridge or gateway between ingroups and outgroups. The gateway group effect is defined as more positive attitudes towards a single-identity outgroup as a result of exposure to a dual-identified group that shares both the ingroup and the outgroup identities [20]. For example, being exposed to immigrants who identify with both their home and host countries can improve attitudes between citizens of each of these two countries [43]. This effect has been studied across several contexts, including minimal groups, the Israeli Palestinian conflict, the Western Balkans, and race relations in the United States [44; 45].

The gateway group effect is further supported by the theoretical perspective of recategorization. The common ingroup identity model [46], a recategorization framework, has proposed that perceiving others as possessing a dual identity can improve attitudes toward an outgroup by seeing the ingroup and outgroup as sharing a common superordinate identity [see also 47]. Thus, the more people perceive a dual-identity group as being associated with the ingroup, the more likely it is that the corresponding outgroup will be incorporated into a superordinate social identity and improve evaluations of the outgroup. These gateway group findings and the related theory or recategorization suggest that the positive reaction to dual identities might “spill over” to improve attitudes towards the corresponding outgroup.

## Research overview

As our World Cup example illustrated, people tend to gravitate to dichotomous, ingroup-outgroup classifications. Furthermore, there is limited research offering insights into the consequences of being exposed to others who are explicitly identified with both one’s ingroup and an outgroup simultaneously. Our core theoretical proposition is that people will express attitudes toward dual-identity groups that are similarly positive to those expressed towards groups who only identify with the ingroup, despite the presence of identification with the outgroup. Drawing on the primary role of ingroup membership in social relations [11], we hypothesize that when people perceive others as explicitly holding a dual identity—which we define both conceptually and empirically as identifying with both identities to a high and relatively similar degree—their perceptions and evaluations will be similar to the ways people perceive and evaluate those who primarily strongly identify with the observer’s ingroup [H1]. For example, we predict that being exposed to Muslim Americans who identify strongly either as American or both as Muslim and as American will produce significantly more positive attitudes towards Muslim Americans than being exposed to Muslim Americans who identify strongly only with their Muslim identity.

Our second hypothesis extends research on the gateway group effect [8] and states that exposure to groups that explicitly identify with both the ingroup and outgroup elements of their dual identity will improve attitudes toward the corresponding outgroup associated with the dual identity [H2]. For example, we expect that being exposed to Muslim Americans who identify strongly with both their Muslim and American identities will lead to more positive attitudes towards Muslims outside America as well.

To understand how perceptions of dual-identification affect attitudes towards dual-identity groups as well as the corresponding outgroup, we conducted three studies using correlational and experimental designs. Study 1 used a correlational design to examine the implications of perceiving Muslim Americans as having a dual identity (i.e., those identified strongly with both their Muslim and American identities) compared to perceiving them as being identified only with the ingroup (American) or only with outgroup identity (Muslims). We correlated perceived dual identity with the attitudes that participants expressed toward (a) Muslim Americans and (b) non-American Muslims (to test the gateway group hypothesis).

Studies 2 and 3 attempted to conceptually replicate Study 1 using an experimental between-subjects design. In Study 2, building on previous work on perceived dual identification [8], we experimentally manipulated the perceived identity of Muslim American as holding either a dual identity (Muslim American), predominantly an ingroup identity (American) or predominantly an outgroup identity (Muslim). We employed this manipulation because past research has found that varying the subjective identification of individuals has a substantial impact on the way they are perceived by others. For example, research in the context of the Arab minority in Israel [8] and the Bosnian minority in Serbia [44] demonstrated that varying the expressed identification of the minority group predicted the perceived identification (by the majority group). Accordingly, Studies 2 and 3 manipulated the expressed identification of the relevant dual identity groups. To increase generalizability, Study 3 was conducted in the context of another dual identity group: Mexican Americans.

Across all three studies we also examined the broader impact of dual identification as a potential gateway to improve intergroup attitudes towards the outgroup that comprised the dual identity. To this end, we tested whether being exposed to a dual-identified group improved attitudes towards the respective single-identity outgroup (non-American Mexicans in Studies 1 and 2, and non-American Muslims in Study 3). Because our core prediction is that perceiving a group as having a dual identification will improve intergroup attitudes, both for the dual-identified group and the corresponding outgroup, we conducted regression analyses in each study with perceived dual identity as the predictor of our intergroup outcomes.

Although we seek to empirically demonstrate that perceiving gateway group members as having a strong dual identity improves intergroup orientations to an extent that is not statistically different from perceiving them as having a strong ingroup identity, we do not wish to claim that the underlying processes are identical. In other words, as perceptions of ingroup identity and dual identity are distinct ways of viewing others, they both can significantly and uniquely improve intergroup relations. While our main hypotheses focus on the claim that perceiving members of a gateway group as having a dual identification would lead to intergroup benefits similar to perceiving them as having a strong identification with the observer’s ingroup, we conducted hierarchical regressions to examine whether ingroup identity and dual identity can both produce meaningful and distinct intergroup benefits. Specifically, we run regressions predicting intergroup attitudes first from perceived outgroup identity, followed by ingroup identity, then by dual identity, in order to demonstrate the unique variance that each perceived identity explains. This order of inclusion in the regression equation allows us to test whether perceived ingroup and dual identity account for significant amounts of variance after considering the effect of outgroup identity, as well as whether perceived dual identity has effects beyond the effects of the two constituent identities (ingroup and outgroup).

To measure intergroup outcomes in a comprehensive manner we examined cognitive perceptions (e.g., stereotypes; [48]), affective responses (e.g., negative group-based feelings; [49]), dehumanization [50], policy support, and resource allocations [51]. We included a measure of threat as well, because recent work has found that perceiving others as having a dual identity can arouse suspicions about their loyalty to the ingroup [42]. Finally, we also measured common ingroup identity because research has found that seeing an outgroup as sharing a common social identity with the ingroup can improve intergroup attitudes [45; 52]. We describe our methods in detail, and make research materials, data, and analysis scripts openly available. Data were analyzed using R, version 4.0.2 [53].

## Study 1: Correlation effects of perceived dual identification

Study 1 used a correlational design to examine the relationship between perceived forms of social identification (ingroup, outgroup, and dual) in the intergroup context of the United States and the Muslim world. We chose this context for two reasons. First, this intergroup context has shaped the global landscape during the past generation and will continue to have a substantial impact for the foreseeable future [54; 55]. Second, compared to the traditional focus of intergroup relations work on racial or ethnic groups, the perceptions of non-Muslim Americans toward Muslims are relatively underrepresented in the literature.

To measure dual identification, we used the dual identity measure by Simon et al. [56]. Our outcome measures of intergroup perception focused on cognitive (stereotypes) and affective reactions (threat and dehumanization). We also measured participants’ perceptions of non-American Muslims to examine the gateway group effect. To minimize demand characteristics, different measures were used to test the attitudes toward the non-American Muslim outgroup. Accordingly, to measure attitudes towards the outgroup, we measured a sense of common identity, group-based feelings, support for aggressive policies against Muslims, and resource allocations. We report the dimensionality for these measures in the Supplementary Materials.

### Method

#### Participants

Prior to data collection, we used G*Power software [57] to determine the target sample size. Based on previous results of correlational studies in the context of intergroup relations and dual identity [8], we aimed to obtain 90% power for the detection of a one-tailed medium effect size (ρ = 0.25) at the standard .05 alpha error probability. Accordingly, the target sample size necessary was 130 participants. Our focus was on responses of non-Muslim participants to Muslims, and, in anticipation of the potential exclusion of Muslim participants from our analyses, we collected data from 150 participants in total. Participants were recruited via MTurk. The recruitment was limited to participants who identified themselves as American, and we verified that the participants were indeed American by using relevant Turkprime filters. Furthermore, we blocked any multiple attempts to participate from the same geolocation as well as locations identified as server farms. Of the full sample, 6 participants were excluded from analyses because they indicated at the end of the survey that they were Muslim, leaving a sample of 144 non-Muslim Americans (86 men, 58 women, 0 other*; M*_*age*_ = 35.49 years, *SD* = 10.52).

#### Design

We measure the extent to which non-Muslim participants perceive Muslim Americans as being identified with the ingroup identity (i.e., American), with the outgroup identity (i.e., Muslim), and with both identities (i.e., having a dual identity). We used a correlational design to examine the implications of perceiving Muslim Americans as having a dual identity, compared to perceiving them as being identified only with the ingroup or outgroup identity, on attitudes toward (a) Muslim Americans and (b) non-American Muslims (to test the gateway group hypothesis).

#### Procedure

Participants were informed that they would be participating in a survey about social issues. After consenting to participate, they completed the following measures in the order in which they are described.

#### Perceived ingroup identity

We assessed the extent to which participants identified Muslim Americans as American (ingroup) by asking participants “to what extent do you feel that Muslim Americans are American,” on a scale ranging from 1 = *not at all* to 100 = *completely* [8].

#### Perceived outgroup identity

We assessed the extent to which participants identified Muslim Americans as Muslims (outgroup) by asking participants “to what extent do you feel that Muslim Americans are Muslim,” on a scale ranging from 1 = *not at all* to 100 = *completely* [8].

#### Perceived dual identity

We assessed the extent to which participants identified Muslim Americans as both American (ingroup) and as Muslims (outgroup) using the dual identity measure by Simon et al. [56]. This direct dual identity measure consists of three items for which participants responded from 1 = *strongly disagree* to 6 = *strongly agree*: “*In my opinion*, *Muslim Americans are both Muslim and American*”; “*Muslim Americans have many Muslim cultural characteristics and many American cultural characteristics*”; and “*Muslim Americans have a lot in common both with Muslims and Americans*” (α = 0.89).

The original measure by Simon et al. [56] includes four items. However, one of the items (“At times, I see Muslim Americans as Muslims and at other times as Americans. It depends on the context”) was negatively correlated with the others, and led to notably low internal consistency (α = 0.62). Removing this item achieved acceptable reliability for the scale for each study. We therefore dropped this item in the measure’s computation in all our studies. Our results are robust to the inclusion of this item.

We also included a secondary measure that assessed perceptions of a dual identity in a more indirect manner. As described in detail in the Supplementary Materials, this measure, rather than asking participants directly about their dual identity perceptions, computed a score based on the relative strengths of gateway group members’ two identity components (in a way mathematically identical to the attitude ambivalence measure created by [58]; see [8]). We found a high correlation in this study between our measure and the more direct and validated measure by Simon et al., [56] (*r*(142) = 0.74, *p* < .001), and the pattern of findings for the two measures were similar. We identify one different, noteworthy result in Study 3.

After the measures of participants’ perceptions of the identities of Muslim Americans, we measured three relevant intergroup perception variables: stereotypes, threat, and dehumanization.

### Intergroup perceptions of dual identity group (Muslim Americans)

#### Stereotypes

Participants completed a stereotypes endorsement measure adapted from Vaes et al. [59]. Specifically, participants indicated the extent to which a number of negative stereotypical traits (*primitive*, *bad*, *dishonest*, *hostile*, *stupid*, and *violent)*, presented in a random order, described Muslim Americans in general (on a scale from 1 = *not at all* to 7 = *completely*; α = 0.95).

#### Threat

To measure whether associating America with Muslims would provoke a sense of threat, we measured threat using the integrated threat measure (ITT) adapted from Levy et al. [44], who based it off Stephan et al. [60] (on a scale from 1 = *strongly disagree* to 6 = *strongly agree*; α = 0.91). This measure includes three items measuring realistic threat (*“Muslim Americans present a risk for increased terror attacks”; “The growing population of Muslim Americans poses a threat to the freedoms of greater American society”; “Police should regard Muslim Americans with extra caution”)* and two items measuring symbolic threat (*“The basic values and beliefs of Muslims and Americans are fairly similar”* (R)*; “Muslim Americans have a negative influence on American society”*).

#### Dehumanization

We measured dehumanization using a single item sentience measure by Leidner et al. [61]: “*To what extent do you estimate that feeling compassion for the suffering of others is a typical trait for Muslim Americans*?” (on a scale from 1 = *not at all typical* to 6 = *very typical*).

### Intergroup perceptions of the outgroup (non-American Muslims): The gateway group effect

We next measured participants’ perceptions toward the respective outgroup–Non-American Muslims–to examine the gateway group effect.

#### Common identity

We measured the sense of a shared or common social identity [51; see also 45] using a three-item measure: “*I see the Muslim world and the Western world as part of one larger cohesive group*;” “*Despite the obvious differences between America and the Muslim world*, *they are part of the same social group*;” and “*Muslims and Americans are two distinct groups with no apparent commonalities”* (R), (on a 1 = *strongly disagree* to 6 = *strongly agree* scale; α = 0.68).

#### Group-based feelings

We measured feelings toward Muslims outside the US, using a single feeling thermometer with responses ranging from 0 = *very negative feelings* to a 100 = *very positive feelings* [62].

#### Support for aggressive policies against Muslims

Next we measured support for aggressive policy toward non-American Muslims using a five-item scale adapted from Levy et al. [8] and based on Sinclair et al. [50]: *“A ban on Muslim immigration”*; *“Rights of Muslim immigrants to maintain their cultural practices across the world such as women’s headscarves”* (R); *“In non-Muslim countries*, *Muslim people’s ID should clearly indicate that they are Muslim”*; *“Strengthening diplomatic ties between the U*.*S*. *and the Muslim world”* (R); and *“The use of more U*.*S*. *military force against insurgence in Muslim countries”* (on a 1 = *very averse* to 6 = *very supportive* scale; α = 0.81).

#### Resource allocation

We devised a “dictator game” resource allocation exercise adapted from previous gateway group effect studies by Levy et al. [8] and based on Tajfel and Turner [7]. In a dictator game, participants have complete control over a resource to be distributed between themselves and others [63]. We adapted the classic dictator game to address three relevant issues for intergroup dynamics: (a) educational resources in the form of allocating a budget for student scholarships between students from the US and students from Muslim countries, (b) resources in the form of allocating Peace Corps volunteer personnel between Muslim and non-Muslim countries, and (c) resources for cultural and religious facilities in the form of allocating a UNESCO restoration budget between ancient mosques and churches. For each of these issues, participants were asked how they think the resources should be allocated between the ingroup and the outgroup (α = 0.68).

After completing all measures, participants provided demographic information including gender, age, and religion.

#### Data analysis

After standardizing all our main predictor and outcome variables, we used multivariate linear regressions to analyze the relationship between perceiving Muslim Americans as being dually identified, identified only with the ingroup, or identified only with the outgroup identity, and multiple measures of intergroup attitudes. Specifically, we conducted hierarchical multivariate linear regressions in which intergroup attitudes (stereotypes, threat, dehumanization, common identity, general feelings, resource allocation, support for aggressive policy) are predicted first from perceived outgroup identity, followed by ingroup identity, then by dual identity, in order to demonstrate the unique variance that each variable explains. We used multivariate regressions rather than running several separate univariate regressions in order to assess the relationship of our predictors with multiple conceptually and empirically related (i.e., correlated) outcomes while controlling for Type I errors. Multivariate regressions assume multivariate normal distribution, linearity, reliability of measurement, and homoscedasticity [64; 65]. The residuals of our regressions were visually inspected using frequency histograms and were found to be normally distributed [65; 66]. Linearity and homoscedasticity were assessed through visual inspection of residual plots and found to be satisfactory [65; 66]. The reliability estimates of all our measures were acceptable.

### Results

#### Dual identity intergroup perception measures

We conducted hierarchical multivariate regression analyses predicting intergroup perceptions (stereotypes, threat, and dehumanization) of the dual-identity group (Muslim Americans), first from perceived outgroup identity (step 1), followed by the addition of perceived ingroup identity as a predictor (step 2), then by the addition of perceived dual identity as a predictor (step 3). The results are presented in Table 1; univariate analyses with all three perceived identity variables included in the model are presented in Table 2. As hypothesized, perceived dual identity predicted less negative perceptions of Muslim Americans, as did perceived ingroup identification, while the effect of perceived outgroup identification on perceptions of Muslim Americans dropped out once perceived ingroup and dual identification were accounted for. These results suggest that both perceived dual identification and perceived ingroup identification (but not perceived outgroup identification) had significantly positive relationships with intergroup attitudes (Table 3).

**Table 1:** Inter-correlations between main variables.

	*1*	*2*	*3*	*4*	*5*	*6*	*7*	*8*	*9*	*10*
*1*. *Ingroup identity*										
*2*. *Outgroup identity*	0.373***									
*3*. *Dual identity*	0.756***	0.314***								
*4*. *Stereotypes*	-0.581***	-0.129	-0.574***							
*5*. *Threat*	-0.648***	-0.221**	-0.678***	0.590***						
*6*. *Dehumanization*	-0.621***	-0.224**	-0.692***	0.552***	0.641***					
*7*. *Common identity*	0.432***	0.182*	0.471***	-0.438***	-0.556***	-0.484***				
*8*. *General feelings*	0.274***	0.186*	0.277***	-0.270**	-0.582***	-0.160	0.204*			
*9*. *Resource allocation*	0.481***	0.122	0.535***	-0.450***	-0.474***	-0.559***	0.534***	0.110		
*10*. *Aggressive policy*	-0.660***	-0.362***	-0.656***	0.518***	0.825***	0.643***	-0.486***	-0.627***	-0.489***	

Computed correlation used Pearson-method with pairwise-deletion.

*p < .05;

**p < .01;

***p < .001

**Table 2:** Hierarchical multivariate regression analysis (Pillai’s trace) with perceived outgroup/ingroup/dual identification predicting intergroup attitudes toward the dual identity group (Muslim Americans): Stereotypes, threat, and dehumanization.

	Model 1 F(3, 140)	Model 2 F(3, 139)	Model 3 F(3, 138)
Outgroup identity	3.06*	0.67	0.86
Ingroup identity		46.19***	7.19***
Dual identity			15.05***
Adjusted R^2^	0.03	0.38	0.46
F-statistic	5.90*	44.21***	41.21***

Adjusted ΔR^2^ = 0.34 for Step 1; Adjusted ΔR^2^ = 0.08 for Step 2

*p < .05;

**p < .01;

***p < .001

**Table 3:** Univariate results with perceived outgroup/ingroup/dual identification predicting intergroup attitudes toward the dual identity group (Muslim Americans).

	Stereotypes	Threat	Dehumanization
Constant	1.67***	1.96***	2.05***
	(0.14)	(0.13)	(0.13)
Outgroup identity	0.18	0.07	0.05
	(0.11)	(0.10)	(0.11)
Ingroup identity	-0.43***	-0.39***	-0.29*
	(0.12)	(0.11)	(0.12)
Dual identity	-0.55**	-0.79***	-0.97***
	(0.17)	(0.16)	(0.17)
Adjusted R^2^	0.38	0.49	0.49
F(3, 140)	30.02***	47.37***	46.99***

Note. Standard errors in parentheses.

*p<0.05;

** p<0.01;

***p<0.001

#### Outgroup perception measures and the gateway group effect

We next tested perceptions of identity predicted attitudes towards the corresponding outgroup (common identity, feelings, aggressive policy, and resource allocation) associated with the dual identity group: i.e., Muslims outside of the United States.

Hierarchical multivariate regression analyses revealed that, as hypothesized, perceived dual identification and perceived ingroup identification both predicted more positive perceptions of non-American Muslims, while the effect of perceived outgroup identification on intergroup perceptions dropped out once perceived ingroup and dual identification were accounted for. Results are presented in Table 4; univariate analyses with all three perceived identity variables included in the model are presented in Table 5. These results suggest that both perceived dual identification and perceived ingroup identification (but not perceived outgroup identification) had significantly positive relationships with intergroup attitudes.

**Table 4:** Hierarchical multivariate regression analysis with perceived outgroup/ingroup/dual identification predicting the intergroup orientations toward the outgroup of non-American Muslims: Common social identity, egalitarian resource allocation, group based negative feelings, and support for aggressive policy.

	Model 1 F(4, 139)	Model 2 F(4, 138)	Model 3 F(4, 137)
Outgroup identity	5.73***	1.57	1.48
Ingroup identity		26.13***	4.21**
Dual identity			6.48***
Adjusted R^2^	0.06	0.26	0.31
F-statistic	10.13**	26.64***	22.18***

Adjusted ΔR^2^ = 0.20 for Step 1; Adjusted ΔR^2^ = 0.04 for Step 2

*p < .05;

**p < .01;

***p < .001

**Table 5:** Univariate regression analysis with perceived outgroup/ingroup/dual identification predicting the intergroup orientations toward the outgroup of non-American Muslims.

	Common identity	General feelings	Aggressive policy	Resource allocation
Constant	0.41***	0.57***	2.00***	0.35**
	(0.11)	(0.12)	(0.12)	(0.11)
Outgroup identity	0.01	0.10	-0.18	-0.10
	(0.09)	(0.10)	(0.10)	(0.09)
Ingroup identity	0.14	0.10	-0.38***	0.18
	(0.09)	(0.10)	(0.10)	(0.10)
Dual identity	0.41**	0.19	-0.58***	0.54***
	(0.14)	(0.15)	(0.15)	(0.14)
Adjusted R^2^	0.22	0.07	0.49	0.29
F(3, 140)	14.34***	4.82**	47.72***	20.52***

Note. Standard errors in parentheses.

*p<0.05;

**p<0.01;

***p<0.001

### Discussion

The results of Study 1 offer a number of important insights. We found support for our hypothesis that perceived dual identity would have a positive impact on intergroup perceptions, both directly toward the dual-identity group (Muslim Americans) but also indirectly toward the corresponding outgroup (non-American Muslims). Overall, Muslim Americans who were perceived to be identified strongly and equally with their Muslim and American identities were viewed positively, as were Muslim Americans who were seen as predominantly identified with America. Muslim Americans perceived to be dually identified also served as a gateway to improve attitudes towards non-Muslim Americans. These data provide a first demonstration that perceived dual identification can offer the benefits of perceived ingroup identity without the detriment of perceived outgroup identity.

## Study 2: Experimental effects of perceived dual identification

Study 2 experimentally manipulated the perceived identification of Muslim Americans. We tested the effects of participants perceiving this group as holding a dual identity (Muslim American) compared to perceiving them as holding predominantly an ingroup identity (American) or predominantly an outgroup identity (Muslim). This experimental design allows for causal identification of dual identity as a driver of intergroup attitudes. After conducting omnibus tests, we conducted the same regression analyses we used in Study 1, given our core proposition that it is perceptions of dual identity that drive intergroup attitudes.

### Method

#### Participants

Based on the effect sizes of Study 1, we aimed for a sample size for Study 2 of 180 that was estimated to be able to detect a medium effect (*f* = 0.25) at 80% power at the standard .05 alpha error probability for a one-way omnibus test with 4 groups [57]. In anticipation of the need to remove participants who failed the comprehension check, we collected data from 200 participants. Of the full sample, 27 participants were excluded because they failed the comprehension check (described below), leaving a sample of 173 non-Muslim Americans (87 men, 75 women, 11 other; *M*_*age*_ = 35.96 years, *SD* = 11.63).

#### Design

We used an experimental between-subjects design to manipulate the perceived identification of Muslim Americans as holding either a dual identity (Muslim American), predominantly an ingroup identity (American), or predominantly an outgroup identity (Muslim). A fourth condition served as a control condition.

#### Experimental manipulation of perceived identification

We manipulated the perceived identification of Muslim American. Participants read an article about Muslim Americans: “An article that was recently published about a survey conducted among Muslim Americans that dealt with the issue of the identity of this population in the USA.” This article described a survey that was conducted regarding their level of identification with each of their social counterparts (i.e., Americans or Muslims, [8; 44]). We manipulated the results of the survey described in the article to create four conditions.

Three of the conditions systematically presented information about how Muslim Americans socially identified; the fourth condition was a control condition in which participants read about an unrelated topic. In each of these three Muslim American identity conditions, the article that participants read described the results of a survey of “more than 1000 Muslims ages 18 and up all over the country,” representing “one of the most thorough surveys on this matter to date.” These three conditions differed in the headline and the purported results.

In the American Identity condition, the headline of an ostensible news article read, “Survey: Majority of Muslim Americans identify mostly with their American identity,” and the text reported that “of the Muslim American participants, 83% claimed that being American plays the most significant role in their personal identity. Hence, the vast majority of Muslim Americans see themselves as primarily American.” The text elaborated on these findings, including corresponding quotes and commentary. (See Supplementary Materials for the complete texts).

In the Muslim Identity condition, the headline indicated, “Survey: Majority of Muslim Americans identify with their Muslim identity,” and reported, in parallel fashion, that “83% claimed that being Muslim plays the most significant role in their personal identity” that “Muslim Americans see themselves primarily as Muslim” and with other text appropriately modified.

In the Dual Identity condition, the headline stated, “Majority of Muslim Americans identify with both of their identities to the same degree.” This article explained that “83% claimed that the USA plays a significant role in their personal identity together with their Muslim identity. Hence, the vast majority of Muslim Americans see themselves as American and at the same time identify as part of the Muslim world.” The text elaborating on these findings was modified in a way that paralleled the other two identity conditions. The article in the fourth condition, the control condition, was of comparable length and format but was about astronomy, a topic not related to the gateway group in any way.

#### Comprehension check measure

After reading the article, as a comprehension check, participants were presented with a multiple-choice question asking, “*What where the conclusions of the survey described in the article*?”, and had to choose one of the following answers: (a) *“The majority of Muslim Americans identifies as both Muslim and American*,*”* (b) *“The majority of Muslim Americans identifies primarily as American*,*” and* (c) *“The majority of Muslim Americans identifies primarily as Muslim*.” Additionally, at the very end of the survey participants were also asked whether they believed that the article they read was true.

As described above, 173 of participants correctly answered the article comprehension questions and believed the article was true. The 27 participants who failed the check were excluded from the analysis. The exclusion of participants did not systematically differ across the three experimental conditions with the article about how Muslim Americans socially identify.

#### Perceived ingroup identity

Participants completed the same perceived identification measures used in Study 1 of American Muslims as American (ingroup) on a 1–100 scale.

#### Perceived outgroup identity

Participants completed the same perceived identification measures used in Study 1 of American Muslims as Muslim (outgroup) on a 1–100 scale.

#### Dual identity measure

Participants completed the same explicit dual identity scale from Study 1 ([56]; α = 0.92). All three perceived identification measures serve both as manipulation checks and as predictor variables in our regression analyses.

#### Intergroup perceptions of dual identity group (Muslim Americans)

To measure perceptions towards Muslim Americans, participants completed the same measures described in Study 1: stereotypes (α = 0.96), threat (α = 0.95), and dehumanization toward the dual identity group.

#### Intergroup perceptions of the outgroup (non-American Muslims): The gateway group effect

To measure perceptions of the outgroup (non-American Muslims), participants complete the same measures used in Study 1: common identity (α = 0.90), group-based feelings, aggressive policy support (α = 0.88), and resource allocation (α = 0.59) toward non-American Muslims. We report the dimensionality for all measures in the Supplementary Materials. See Table 6 for inter-correlations.

**Table 6:** Inter-correlations between main variables.

	*1*	*2*	*3*	*4*	*5*	*6*	*7*	*8*	*9*	*10*
*1*. *Ingroup identity*	-									
*2*. *Outgroup identity*	0.136	-								
*3*. *Dual identity*	0.715***	0.126	-							
*4*. *Stereotypes*	-0.629***	-0.007	-0.636***	-						
*5*. *Threat*	-0.730***	-0.076	-0.738***	0.789***	-					
*6*. *Dehumanization*	-0.573***	0.000	-0.624***	0.662***	0.715***	-				
*7*. *Common identity*	0.613***	0.074	0.690***	-0.660***	-0.791***	-0.680***	-			
*8*. *General feelings*	0.656***	0.007	0.668***	-0.808***	-0.803***	-0.691***	0.715***	-		
*9*. *Resource allocation*	0.417***	-0.079	0.445***	-0.538***	-0.538***	-0.511***	0.508***	0.607***	-	
*10*. *Aggressive policy*	-0.723***	-0.107	-0.731***	0.728***	0.881***	0.677***	-0.742***	-0.715***	-0.516***	-

Computed correlation used Pearson-method with pairwise-deletion.

*p < .05;

**p < .01;

***p < .001

#### Data analysis

First, we standardized all our main predictor and outcome variables, and then performed MANOVAs to test the effect of the experimental condition on perceived identification. We then used multivariate linear regressions to test the effects of the experimental condition on the various measures of intergroup attitudes (stereotypes, threat, dehumanization, common identity, general feelings, resource allocation, and support for aggressive policy). We also conducted these analyses with the manipulation conditions dummy coded in order to probe the group comparisons. We set the dual identity group as the reference group, to test our predictions that the effect of the dual identity group will not be significantly different from the effect of the ingroup identity group, and will be significantly different from the effect of the outgroup identity group and the control group. We used MANOVAs and multivariate linear regressions rather than running several separate univariate regressions in order to assess the effect of the experimental condition on multiple conceptually and empirically related (i.e., correlated) outcomes while controlling for Type I errors. The residuals of our regressions were visually inspected using frequency histograms and were found to be normally distributed [65; 66]. Linearity and homoscedasticity were assessed through visual inspection of residual plots and found to be satisfactory [65; 66]. The reliability estimates of all our measures were acceptable.

Next, we performed the same hierarchical multivariate regression analyses we used in Study 1, given our core proposition that it is perceptions of dual identity that drive intergroup attitudes.

Finally, again setting dual identity as the reference group, we ran mediation analyses testing the indirect effect of the identity manipulation through perceived ingroup identity on our outcomes. Mediation analyses assume no unmeasured confounding of the treatment-outcome relationship, no unmeasured mediator-outcome confounding, and no unmeasured treatment-mediator confounding. The first and third assumptions are satisfied by the random assignment of our manipulation. However, because only the treatment variable was randomly assigned, and not the mediator, we avoid making causal mediation claims.

### Results

#### Dual identity measure

**Fig 1:**
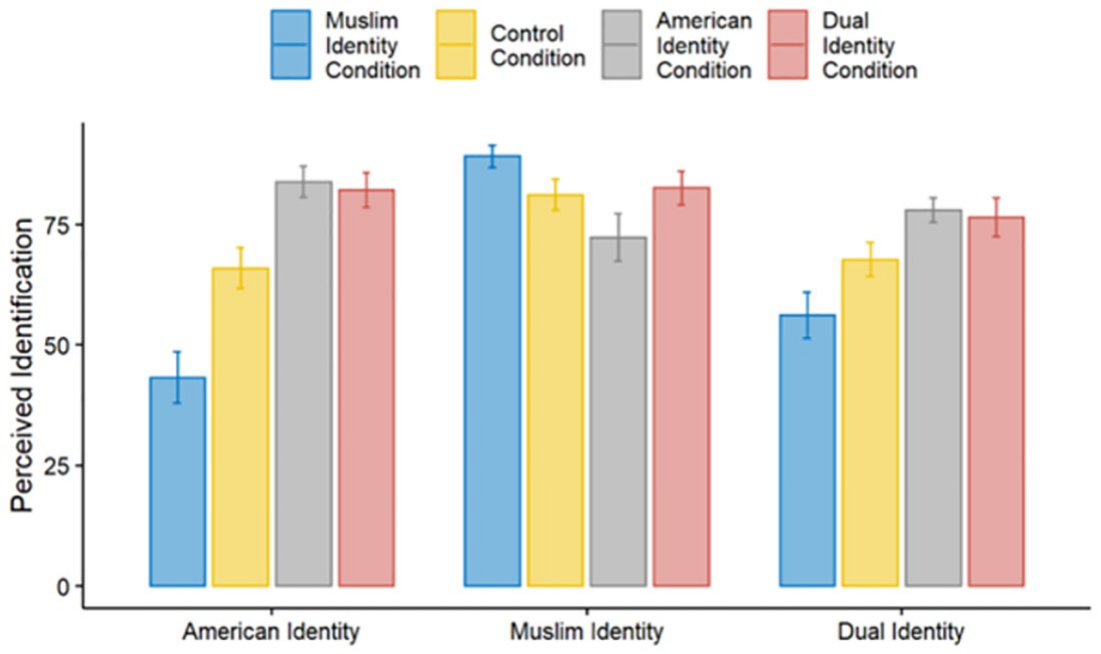
Perceived identification of Muslim Americans with both American and Muslim identities and the dual identity measure across all four conditions. The dual identity measure is scaled to a 0–100 scale for presentation purposes. Error bars indicate standard errors.

Our dual identity measure serves as both a manipulation check and as a predictor variable in our regression analyses. We first established that the manipulation had the expected effect. The experimental manipulation had a significant impact on perceived dual identity, *F*(3, 169) = 5.91, *p* < 0.001. Participants in the Dual Identity condition perceived Muslim Americans as having a stronger dual identity than participants in the Muslim Identity condition and a marginally stronger dual identity than participants in the Baseline Control condition, though they did not differ significantly from participants in the American Identity condition (Table 7, Model 1). There was also a significant effect of experimental condition on perceptions that Muslim Americans identified as American, *F*(3, 169) = 16.52, *p* < .001, with participants in the Dual Identity condition and the American Identity condition seeing Muslim Americans as more identified as American compared to the Baseline Control condition (Table 7, Models 4 and 5, respectively), which in turn scored higher than the Muslim Identity condition (Table 7, Model 6). Finally, the experimental manipulation affected perceptions Muslim Americans identified as Muslims, *F*(3, 169) = 3.09, *p* = .029. Participants in the Muslim Identity condition perceived Muslim Americans as most strongly identifying with being Muslim, but they did not differ significantly from participants in the Dual Identity or in the Baseline Control conditions (Table 7, Model 9); participants in the American Identity condition, however, did perceive Muslim Americans as being marginally or significantly less identified as Muslim than did participants in each of the other three conditions (Table 7, Model 8). See Fig 1 for means by condition.

**Table 7:** Comparison across manipulation conditions of perceived identification of Muslim Americans with both American and Muslim identities.

	Dual identity			Ingroup identity			Outgroup identity		
	(1)	(2)	(3)	(4)	(5)	(6)	(7)	(8)	(9)
Constant	1.03***	1.05***	0.81***	1.08***	1.10***	0.57***	0.97***	0.85***	1.05***
	(0.04)	(0.04)	(0.05)	(0.06)	(0.06)	(0.06)	(0.04)	(0.05)	(0.05)
Muslim identity condition dummy	-0.22***	-0.23***		-0.51***	-0.53***		0.08	0.20**	
	(0.06)	(0.06)		(0.09)	(0.09)		(0.06)	(0.07)	
American identity condition dummy	0.02		0.23***	0.02		0.53***	-0.12.		-0.20**
	(0.06)		(0.06)	(0.08)		(0.09)	(0.06)		(0.07)
Dual identity condition dummy		-0.02	0.22***		-0.02	0.51***		0.12.	-0.08
		(0.06)	(0.06)		(0.08)	(0.09)		(0.06)	(0.06)
Control condition dummy	-0.09.	-0.11.	0.12*	-0.21**	-0.24**	0.30***	-0.02	0.10.	-0.09
	(0.05)	(0.06)	(0.06)	(0.07)	(0.08)	(0.08)	(0.06)	(0.06)	(0.06)
Adjusted R^2^	0.08			0.21			0.04		
F(3, 169)	5.91***			16.52***			3.09*		

Note. Standard errors in parentheses.

.p<0.1;

*p<0.05;

** p<0.01;

***p<0.001

#### Intergroup perceptions of dual identity group

Using MANOVAs, we tested the effect of the experimental manipulation on our three intergroup perception measures of the dual identity Muslim American group: stereotypes, threat, and dehumanization.

**Fig 2:**
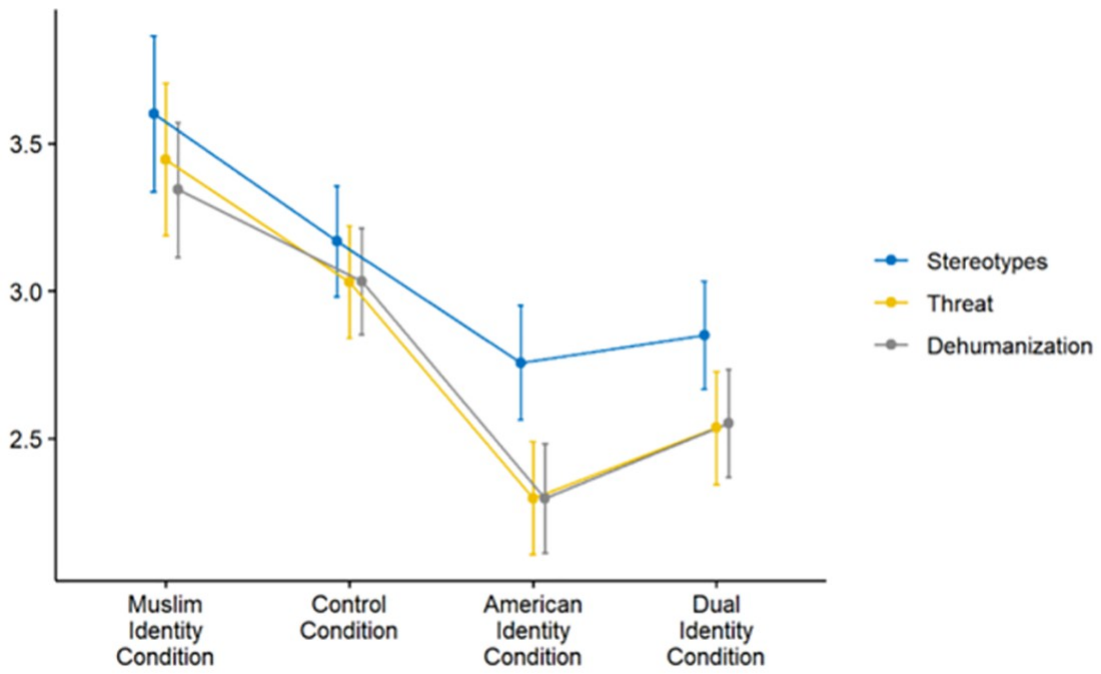
Attitudes toward Muslim Americans based on perceived identification across all four conditions. Error bars indicate standard errors.

There was a significant effect on intergroup perceptions of Muslims Americans, *F*(9, 507) = 2.09, *p* = .029. Participants in the Muslim Identity condition had more negative attitudes towards Muslim Americans than in the American Identity (*F*(3, 167) = 4.90, *p* = .003) and Dual Identity (*F*(3, 167) = 3.01, *p* = .032) conditions, which did not differ from each other (*F*(3, 167) = 0.38, *p* = .769; see Fig 2 for means by condition). Finally, hierarchical multivariate regressions using the perceived identity measures found that perceived dual identity predicted less negative perceptions of Muslim Americans, as did perceived ingroup identification, while the effect of perceived outgroup identification on perceptions of Muslim Americans dropped out once perceived ingroup and dual identification were accounted for. These results suggest that perceived dual identification and perceived ingroup identification both had significantly positive effects on intergroup perceptions. See Table 8 for hierarchical regression results and Table 9 for univariate analyses.

**Table 8:** Hierarchical multivariate regression analysis with perceived outgroup/ingroup/dual identification predicting the intergroup attitudes toward the dual identity group (Muslim Americans): Stereotypes, threat, and dehumanization.

	Model 1 F(3, 169)	Model 2 F(3, 168)	Model 3 F(3, 167)
Outgroup identity	0.97	0.97	1.29
Ingroup identity		66.53***	13.34***
Dual identity			17.17***
Adjusted R^2^	-0.003	0.42	0.51
F-statistic	0.42	63.57***	60.28***

Adjusted ΔR^2^ = 0.42 for Step 1; Adjusted ΔR^2^ = 0.09 for Step 2

*p < .05;

**p < .01;

***p < .001

**Table 9:** Univariate regression analysis with perceived outgroup/ingroup/dual identification predicting the intergroup attitudes toward the dual identity group (Muslim Americans).

	Stereotypes	Threat	Dehumanization
Constant	1.65***	1.93***	1.67***
	(0.11)	(0.10)	(0.12)
Outgroup identity	0.13	0.06	0.14
	(0.08)	(0.07)	(0.09)
Ingroup identity	-0.36***	-0.45***	-0.27**
	(0.08)	(0.07)	(0.08)
Dual identity	-0.57***	-0.71***	-0.68***
	(0.12)	(0.11)	(0.13)
Adjusted R^2^	0.47	0.62	0.42
F(3, 169)	50.94***	95.48***	42.67***

Note. Standard errors in parentheses.

*p<0.05;

** p<0.01;

***p<0.001

#### Intergroup perceptions of the outgroup: The gateway group effect

We next tested the effects of our perceived identity manipulation on perceptions of non-American Muslims using the same measures as Study 1: common identity, intergroup feelings, resource allocation, and support for aggressive policy.

**Fig 3:**
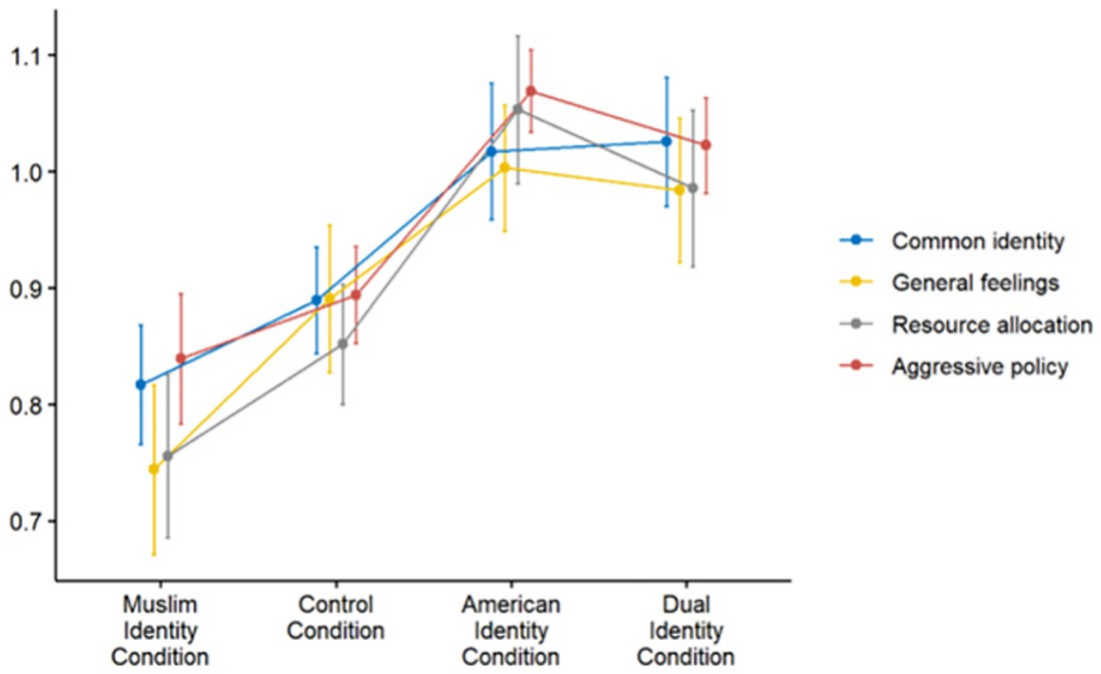
Attitudes toward non-American Muslims based on perceived identification across all four conditions (values were normalized by the root mean square, and aggressive policy was reverse coded, for presentation purposes).

As expected, a MANOVA revealed a significant effect on intergroup attitudes towards non-American Muslims, *F*(12, 471) = 1.80, *p* = .045. Consistent with predictions, participants in the Muslim Identity condition displayed more negative attitudes towards non-American Muslims than in the American Identity (*F*(4, 155) = 3.73, *p* = .006) and Dual Identity (*F*(4, 155) = 2.44, *p* = .049) conditions, which did not differ from each other (*F*(4, 155) = 0.49, *p* = .740; see Fig 3 for normalized means by condition). Finally, hierarchical multivariate regressions using our identity measures found that perceived dual identification, as well as perceived ingroup identification, predicted more positive attitudes towards non-American Muslims. In contrast, the effect of perceived outgroup identification dropped out once perceived ingroup and dual identification were accounted for. These results suggest that both perceived dual identification and perceived ingroup identification had significantly positive effects on perceptions of non-American Muslims. See Table 10 for hierarchical regression results and Table 11 for univariate analyses.

**Table 10:** Hierarchical multivariate regression analysis with perceived outgroup/ingroup/dual identification predicting the intergroup orientations toward the outgroup of non-American Muslims: Common social identity, egalitarian resource allocation, group based negative feelings, and support for aggressive policy.

	Model 1 F(4, 157)	Model 2 F(4, 156)	Model 3 F(4, 155)
Outgroup identity	1.50	1.28	1.48
Ingroup identity		50.13***	10.62***
Dual identity			17.61***
Adjusted R^2^	0.0002	0.39	0.48
F-statistic	1.04	51.41***	50.62***

Adjusted ΔR^2^ = 0.38 for Step 1; Adjusted ΔR^2^ = 0.10 for Step 2

*p < .05;

**p < .01;

***p < .001

**Table 11:** Univariate regression analysis with perceived outgroup/ingroup/dual identification predicting the intergroup orientations toward the outgroup of non-American Muslims.

	Common identity	General feelings	Aggressive policy	Resource allocation
Constant	0.17	0.09	1.96***	0.42***
	(0.09)	(0.11)	(0.10)	(0.13)
Outgroup identity	-0.07	-0.11	-0.03	-0.20*
	(0.07)	(0.08)	(0.08)	(0.10)
Ingroup identity	0.19**	0.37***	-0.43***	0.20*
	(0.07)	(0.08)	(0.07)	(0.10)
Dual identity	0.70***	0.62***	-0.67***	0.55***
	(0.09)	(0.12)	(0.11)	(0.14)
Adjusted R^2^	0.53	0.51	0.61	0.27
F(3, 158)	62.70***	56.93***	85.99***	20.64***

Note. Standard errors in parentheses.

*p<0.05;

** p<0.01;

***p<0.001

#### Mediation analyses

To provide further support of our hypothesis that the ingroup component of the perceived dual identity leads to a positive impact on intergroup orientations despite the presence of the outgroup identity component, we also tested the indirect effect of the identity manipulation through perceived ingroup identity on our outcomes. We employed a mediation analysis, setting the Dual Identity condition as the reference group, perceived ingroup identity as a mediator, and intergroup attitudes as dependent variables, and using 5000 bootstraps [67]. As expected, we found that the indirect effect was significant for all measures for the comparison between the dual-identity condition with the outgroup and control conditions (see Table 12). Also as predicted, the indirect effect was non-significant in all measures for the comparison with the ingroup condition. In other words, the dual identity condition led to similar perceived ingroup identification as the ingroup identity condition, and it was this increase in perceived ingroup identification which improved intergroup attitudes.

**Table 12:** Mediation analyses of the effects of the identity manipulation on intergroup attitudes through perceived ingroup identification, comparing to the dual identity condition.

Comparison Group	Dependent Variable	Direct Effect	Indirect Effect	Total Effect	Adjusted R2
Ingroup condition	Stereotypes	-0.01 [-0.13, 0.11]	-0.02 [-0.10, 0.07]	-0.03 [-0.18, 0.13]	0.39
	Threat	-0.06 [-0.16, 0.05]	-0.02 [-0.12, 0.08]	-0.07 [-0.24, 0.09]	0.53
	Dehumanization	-0.07 [-0.2, 0.07]	-0.01 [-0.09, 0.06]	-0.08 [-0.24, 0.08]	0.32
	Common identity	-0.02 [-0.15, 0.11]	0.01 [-0.05, 0.08]	-0.01 [-0.16, 0.15]	0.37
	General feelings	0.00 [-0.12, 0.12]	0.02 [-0.07, 0.11]	0.02 [-0.14, 0.18]	0.43
	Resource allocation	0.06 [-0.1, 0.23]	0.01 [-0.04, 0.06]	0.07 [-0.11, 0.26]	0.16
	Aggressive policy	-0.05 [-0.14, 0.04]	-0.02 [-0.12, 0.08]	-0.07 [-0.23, 0.09]	0.53
Outgroup condition	Stereotypes	-0.12 [-0.28, 0.03]	0.34*** [0.22, 0.48]	0.22*** [0.04, 0.41]	0.39
	Threat	-0.14 [-0.30, 0.03]	0.42*** [0.28, 0.59]	0.29** [0.09, 0.48]	0.53
	Dehumanization	-0.05 [-0.22, 0.13]	0.3*** [0.17, 0.45]	0.25** [0.07, 0.44]	0.32
	Common identity	0.07 [-0.06, 0.21]	-0.28*** [-0.4, -0.18]	-0.21** [-0.36, -0.06]	0.37
	General feelings	0.13 [-0.03, 0.29]	-0.37*** [-0.51, -0.24]	-0.24* [-0.43, -0.06]	0.43
	Resource allocation	-0.03 [-0.25, 0.17]	-0.2*** [-0.33, -0.09]	-0.23* [-0.42, -0.05]	0.16
	Aggressive policy	-0.14 [-0.30, 0.03]	0.41*** [0.27, 0.56]	0.27** [0.07, 0.47]	0.53
Control condition	Stereotypes	-0.05 [-0.16, 0.07]	0.14** [0.05, 0.24]	0.09 [-0.05, 0.25]	0.39
	Threat	-0.02 [-0.13, 0.09]	0.18** [0.06, 0.29]	0.16 [-0.01, 0.32]	0.53
	Dehumanization	0.03 [-0.10, 0.17]	0.12** [0.04, 0.22]	0.15 [0.00, 0.31]	0.32
	Common identity	-0.02 [-0.14, 0.10]	-0.12** [-0.20, -0.04]	-0.14 [-0.28, 0.00]	0.37
	General feelings	0.07 [-0.06, 0.20]	-0.17** [-0.28, -0.05]	-0.09 [-0.26, 0.08]	0.43
	Resource allocation	-0.05 [-0.21, 0.11]	-0.08** [-0.16, -0.03]	-0.13 [-0.29, 0.03]	0.16
	Aggressive policy	0.01 [-0.10, 0.12]	0.18** [0.07, 0.30]	0.19* [0.02, 0.36]	0.53

Note. 95% confidence intervals in brackets.

*p<0.05;

** p<0.01;

***p<0.001

### Discussion

Study 2 replicated the findings from Study 1 using an experimental manipulation of perceived identity. Specifically, we manipulated whether Muslim-Americans were perceived as identified with their American identity, their Muslim identity, or both identities; we also included a baseline condition. Perceiving a group as having a dual identity (e.g., identified as both American and Muslim) had a similar positive impact on intergroup attitudes as perceiving that group as only identified with the ingroup (Americans) and the opposite impact from perceiving them as identified with the outgroup (Muslims). Furthermore, our experimental manipulation also produced results consistent with the gateway effect, where the manipulation of dual identification improved intergroup attitudes toward non-American Muslims. Across all the measures, we found support for our predicted pattern that the perceived dual identity condition would produce attitudes similarly positive to those in the perceived ingroup identity condition and more positive than those in the baseline condition, which would be higher than those in the perceived outgroup identity condition. Additionally, we directly replicated the results of Study 1 by using perceived dual identity as a predictor of our intergroup attitude measures. Finally, our mediation analyses provided support for the indirect effect of the identity manipulation through perceived ingroup identity on our outcomes. These findings provide further support to our hypothesis that attitudes toward people seen as holding a dual identity are positive because of the ingroup component of the perceived dual identity despite the presence of the outgroup identity component.

## Study 3: Generalizing the effects of perceived dual identity to Mexican Americans

Study 3 was designed to extend our results in two ways. First, we sought to provide generalizability by using a new intergroup context, that of Mexican Americans. We selected Mexican Americans as our dual-identity group based on a pilot study we conducted comparing dual-identity perception of different minority groups in the United States (see supplemental material for more details). Exploring perceptions of Mexican Americans also generalizes our research to dual identities comprised of two identities on the same dimension (national identity) versus two identities from potentially different dimensions (national and religious/ethnicity of Muslim Americans). Second, we measured perceived ingroup and outgroup identification at two separate time points—two weeks before and right after our identification manipulation. This experimental design allowed us to control for baseline variance in perceptions of identification. Similar to Study 2, after conducting omnibus tests, we also conducted the same regression analyses we used in Studies 1 and 2 to confirm our core premise that it is perceptions of dual identity that drive intergroup attitudes.

### Method

#### Participants

Based on the results of Study 1 and 2 we aimed for a sample size for Study 3 of 326 that was estimated to be able to detect a small-medium effect (f^2^ = 0.25) at 90% power at the standard .05 alpha error probability for an omnibus test with 4 groups and one covariate [57]. Due to the anticipated attrition of approximately 20% between the two time points, in the first round (T1) we recruited 475 American participants (244 *men*, 228 *women*, 3 *other; M*_*age*_ = 36.63 *years*, *SD* = 11.32) via Mturk, and in the second round two weeks later (T2) we recruited 368 participants out of the pool of participants that completed the survey in T1 (198 *men*, 165 *women*, 5 *other; M*_*age*_ = 37.97 *years*, *SD* = 11.79). In T1 all participants filled out the same survey, and in T2 the participants were randomly assigned to different identification conditions in a similar manner to Study 2.

#### Design

In T2 we used an experimental between-subjects design, as in Study 2, to manipulate the perceived identification of Mexican Americans as holding either a dual identity (Mexican American), predominantly an ingroup identity (American), or predominantly an outgroup identity (Mexican). We measured perceived identification in two separate time points, both before and after the manipulation of expressed identification.

#### Time 1 perceived ingroup identity

At T1, we measured perceived identification of Mexican Americans as American (ingroup) on a 1–100 scale, using the same measure as in Studies 1 and 2.

#### Time 1 perceived outgroup identity

At T1, we measured perceived identification of Mexican Americans as Mexican (outgroup) on a 1–100 scale, using the same measure as in Studies 1 and 2.

#### Time 2 manipulation

Two weeks later at T2, the same participants read articles similar to those from Study 2. The article described a survey that was conducted regarding the level of identification that Mexican Americans had with being American and Mexican and randomly varied whether the article described American identity, Mexican identity, dual identity, or a control condition.

#### Time 2 perceived ingroup identity

Participants next answered the same perceived identification measure from T1 of Mexican Americans as American (ingroup) on a 1–100 scale.

#### Time 2 perceived outgroup identity

Participants also answered the same perceived identification measure from T1 of Mexican Americans as Mexican (outgroup) on a 1–100 scale.

#### Time 2 perceived dual identity

Participants also completed the dual identity scale from Studies 1 and 2 (Simon et al., 2013; α = 0.87). These perceived identification measures serve both as manipulation checks and as the predictor variables in our regression analyses.

As mentioned in Study 1 and described in further detail in the Supplementary Materials, we also included a secondary measure that assessed perceptions of a dual identity in a more indirect manner. We found a moderate correlation in this study between our measure and the measure by Simon et al., [56] (*r*(364) = 0.62, *p* < .001). Because we measured perceived ingroup and outgroup identification of Mexican Americans at two time points, we can examine change in our secondary measure (which is calculated using these two components) from T1 to T2.

#### Time 2 intergroup perceptions of dual identity group (Mexican Americans)

To measure perceptions towards Mexican Americans, participants completed the same measures described in Study 1: stereotypes (α = 0.96), threat (α = 0.90), and dehumanization toward the dual identity group.

#### Time 2 intergroup perceptions of the outgroup (non-American Mexicans: The gateway group effect

To examine the gateway group effect, participants reported their intergroup attitudes toward the respective outgroup, non-American Mexicans: common identity (α = 0.78), group-based feelings, aggressive policy support (α = 0.89), and resource allocation (α = 0.78). We report the dimensionality for these measures in the Supplementary Materials. See Table 13 for inter-correlations.

**Table 13:** Inter-correlations between main variables.

	*1*	*2*	*3*	*4*	*5*	*6*	*7*	*8*	*9*	*10*	*11*	*12*
*1*. *T1 Ingroup identity*	-											
*2*. *T1 Outgroup identity*	-0.028	-										
*3*. *T2 Ingroup identity*	0.620***	-0.047	-									
*4*. *T2 Outgroup identity*	0.048	0.445***	0.018	-								
*5*. *Dual identity*	0.481***	0.173**	0.535***	0.310***	-							
*6*. *Stereotypes*	-0.461***	-0.010	-0.538***	0.006	-0.479***	-						
*7*. *Threat*	-0.537***	0.016	-0.629***	-0.023	-0.577***	0.718***	-					
*8*. *Dehumanization*	-0.486***	0.000	-0.532***	-0.049	-0.538***	0.527***	0.602***	-				
*9*. *Common identity*	0.388***	0.076	0.453***	0.144**	0.552***	-0.457***	-0.533***	-0.507***	-			
*10*. *Resource allocation*	0.188***	-0.004	0.254***	0.115*	0.309***	-0.237***	-0.379***	-0.311***	0.380***	-		
*11*. *General feelings*	0.411***	-0.052	0.464***	-0.004	0.376***	-0.599***	-0.732***	-0.369***	0.367***	0.212***	-	
*12*. *Aggressive policy*	-0.461***	0.016	-0.540***	-0.098	-0.526***	0.579***	0.809***	0.504***	-0.519***	-0.452***	-0.675***	-

Computed correlation used Pearson-method with pairwise-deletion.

*p < .05;

**p < .01;

***p < .001

#### Data analysis

First, we standardized all our main predictor and outcome variables, and then conducted an MANOVA to test the effect of the experimental condition on perceived dual identification at T2, controlling for perceived ingroup and outgroup identification at T1. We then used multivariate linear regressions to test the effects of the experimental condition on the various measures of intergroup attitudes (stereotypes, threat, dehumanization, common identity, general feelings, resource allocation, and support for aggressive policy). We also ran these analyses with the manipulation conditions dummy coded, setting the dual identity group as the reference group as in Study 2, in order to probe the group comparisons. We used MANOVAs and multivariate linear regressions rather than running several separate univariate regressions in order to assess the effect of the experimental condition on multiple conceptually and empirically related (i.e., correlated) outcomes while controlling for Type I errors. The residuals of our regressions were visually inspected using frequency histograms and were found to be normally distributed [65; 66]. Linearity and homoscedasticity were assessed through visual inspection of residual plots and found to be satisfactory [65; 66]. The reliability estimates of all our measures were acceptable.

Next, we performed the same hierarchical multivariate regression analyses we used in Studies 1 and 2, given our core proposition that it is perceptions of dual identity that drive intergroup attitudes.

Finally, again setting dual identity as the reference group, we employed a mediation analysis to test the indirect effect of the identity manipulation through perceived ingroup identity on our outcomes. (As in Study 2, we avoid claiming causal mediation.) All analyses were conducted controlling for perceived ingroup and outgroup identification in T1.

### Results

#### Dual identity measure

**Fig 4:**
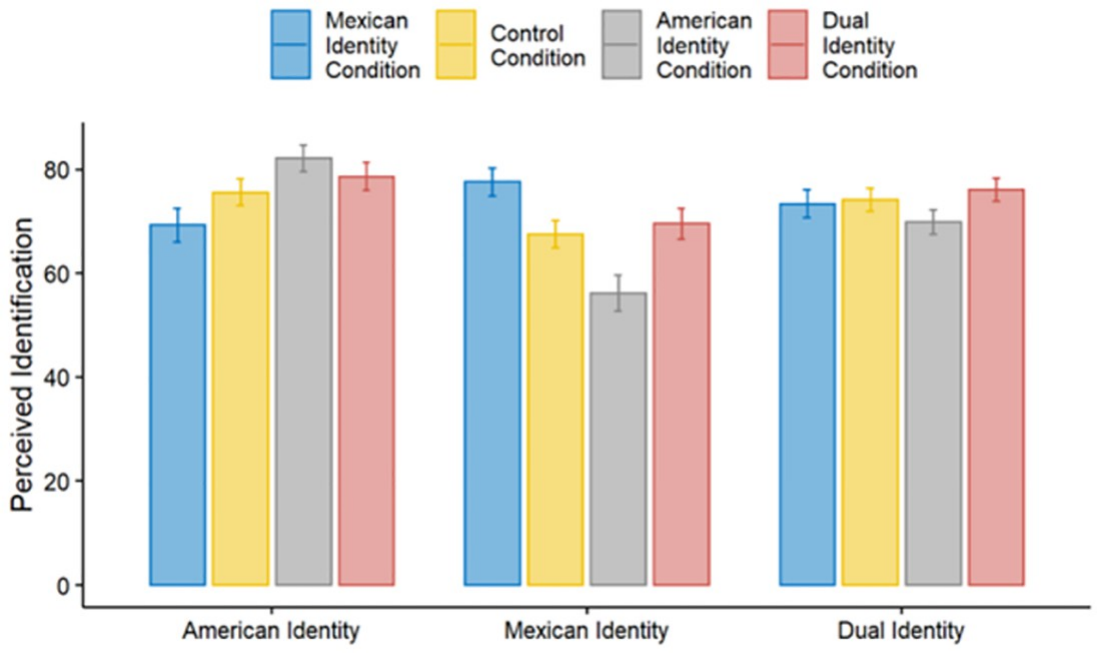
Perceived identification at T2 of Mexican Americans with both American and Mexican identities and the dual identity measure across all four conditions. The dual identity measure is scaled to a 0–100 scale for presentation purposes. Error bars indicate standard errors.

As in Study 2, our dual identity measure both serves as a manipulation check and as a predictor variable. We first tested whether our manipulation of perceived identity altered perceptions of Mexican Americans’ identity. As expected, participants in the dual identity condition displayed greater perceptions of Mexican-Americans’ dual identity than in the perceived ingroup identification condition and the perceived outgroup identification condition, though these differences did not reach significance, *F*(3, 350) = 1.77, *p* = .152 (see Fig 4).

However, using our secondary measure of perceived dual identity, we found that participants in the dual identity condition displayed the biggest *increase* in perceptions of Mexican-Americans’ dual identity (comparing timepoints before and after the manipulation), compared to all other conditions, both with (*F*(3, 350) = 3.37, *p* = .019) and without (*F*(3, 352) = 4.12, *p* = .007) controlling for perceptions of identity at T1.

There was also a significant effect of experimental condition on perceptions that Mexican Americans identified as American, *F*(3, 350) = 11.56, *p* < .001, with participants in the Dual Identity condition seeing Mexican Americans as more identified as American compared to the Mexican Identity condition (*F*(1, 350) = 17.39, *p* < .001), though they did not differ significantly from participants in the American Identity (*F*(1, 350) = 2.36, *p* = .125) or the control (*F*(1, 350) = 2.06, *p* = .152) conditions. Finally, the experimental manipulation affected perceptions Mexican Americans identified as Mexican, *F*(3, 350) = 7.63, *p* < .001, with participants in the American Identity condition perceiving Mexican Americans as least strongly identifying with being Mexican, compared to the other three conditions (*F*_*dual*_(1, 350) = 12.85, *p* < .001; *F*_*control*_(1, 350) = 5.41, *p* = .021; *F*_*Muslim*_(1, 350) = 20.06, *p* < .001).

#### Intergroup perceptions of dual identity group (Mexican Americans)

We first tested whether there was a significant effect of the manipulation on our three intergroup perception measures of the dual identity Mexican American group (stereotypes, threat, and dehumanization), using MANOVAs and controlling for perceived identification in T1. The effect was significant, *F*(9, 1050) = 1.95, *p* = .043. Participants in the Mexican Identity condition had more negative attitudes towards Mexican Americans than in the American Identity condition (*F*(3, 348) = 3.04, *p* = .029) and directionally more negative attitudes than in the Dual Identity condition (*F*(3, 348) = 2.02, *p* = .111), which did not consistently differ from each other (*F*(3, 348) = 1.67, *p* = .174).

Finally, hierarchical multivariate regressions using the perceived identity measures and controlling for perceived identification in T1 found that perceived dual identity predicted less negative perceptions of Mexican Americans, as did perceived ingroup identification, while the effect of perceived outgroup identification on perceptions of Mexican Americans dropped out once perceived ingroup and dual identification were accounted for. These results suggest that both perceived dual identification and perceived ingroup identification had significantly positive effects on perceptions of Mexican Americans. See Table 14 for hierarchical regression results and Table 15 for univariate results.

**Table 14:** Hierarchical multivariate regression analysis with perceived outgroup/ingroup/dual identification predicting the intergroup attitudes toward the dual identity group (Mexican Americans): Stereotypes, threat, and dehumanization.

	Model 1 F(3, 350)	Model 2 F(3, 349)	Model 3 F(3, 348)
Outgroup identity (T2)	0.91	0.91	2.44
Ingroup identity (T2)		33.35***	16.16***
Dual identity (T2)			23.68***
Ingroup identity (T1)	yes	yes	yes
Dual identity (T1)	yes	yes	yes
Adjusted R^2^	0.24	0.35	0.41
F-statistic	38.46***	48.60***	50.36***

Adjusted ΔR^2^ = 0.11 for Step 1; Adjusted ΔR^2^ = 0.06 for Step 2

*p < .05;

**p < .01;

***p < .001

**Table 15:** Univariate regression analysis with perceived outgroup/ingroup/dual identification predicting the intergroup attitudes toward the dual identity group (Mexican Americans).

	Stereotypes	Threat	Dehumanization
Constant	1.91***	2.04***	1.89***
	(0.09)	(0.08)	(0.08)
Outgroup identity (T2)	0.15*	0.10*	0.06
	(0.06)	(0.05)	(0.05)
Ingroup identity (T2)	-0.42***	-0.44***	-0.27***
	(0.08)	(0.07)	(0.07)
Dual identity (T2)	-0.55***	-0.65***	-0.60***
	(0.11)	(0.09)	(0.09)
Outgroup identity (T1)	-0.04	0.02	0.02
	(0.06)	(0.05)	(0.05)
Ingroup identity (T1)	-0.19**	-0.21***	-0.22***
	(0.07)	(0.06)	(0.06)
Adjusted R^2^	0.36	0.49	0.38
F(5, 350)	40.83***	69.20***	44.58***

Note. Standard errors in parentheses.

*p<0.05;

** p<0.01;

***p<0.001

#### Intergroup perceptions of the outgroup (non-American Mexicans): The gateway group effect

We next tested whether dual identification would have similar effects on the corresponding outgroup of non-American Mexicans. A MANOVA revealed a significant effect on intergroup attitudes, controlling for perceived identification in T1, *F*(12, 1077) = 2.23, *p* = .009. Attitudes held by participants in the Dual Identity condition differed from those held by participants in the Mexican Identity condition (*F*(4, 347) = 2.02, *p* = .092) and in the American Identity condition (*F*(4, 347) = 5.03, *p* < .001), though they did not vary consistently across measures.

Finally, a hierarchical multivariate regression using the perceived identity measures and controlling for perceived identification in T1 found that perceived dual identity predicted less negative perceptions of non-American Mexicans, as did perceived ingroup identification, while the effect of perceived outgroup identification on intergroup perceptions dropped out once perceived ingroup and dual identification were accounted for. These results suggest that both perceived dual identification and perceived ingroup identification had significantly positive effects on perceptions of non-American Mexicans. See Table 16 for hierarchical regression results and Table 17 for univariate analyses.

**Table 16:** Hierarchical multivariate regression analysis with perceived outgroup/ingroup/dual identification predicting the intergroup orientations toward the outgroup of non-American Mexicans: Common social identity, egalitarian resource allocation, group based negative feelings, and support for aggressive policy.

	Model 1 F(4, 349)	Model 2 F(4, 348)	Model 3 F(4, 347)
Outgroup identity (T2)	2.52*	2.66*	0.79
Ingroup identity (T2)		17.23***	7.54***
Dual identity (T2)			16.92***
Ingroup identity (T1)	yes	yes	yes
Dual identity (T1)	yes	yes	yes
Adjusted R^2^	0.16	0.22	0.27
F-statistic	22.77***	26.39***	27.72***

Adjusted ΔR^2^ = 0.07 for Step 1; Adjusted ΔR^2^ = 0.05 for Step 2

*p < .05;

**p < .01;

***p < .001

**Table 17:** Univariate regression analysis with perceived outgroup/ingroup/dual identification predicting the intergroup orientations toward the outgroup of non-American Mexicans.

	Common identity	General feelings	Aggressive policy	Resource allocation
Constant	0.27***	0.56***	2.02***	0.38***
	(0.06)	(0.05)	(0.10)	(0.10)
Outgroup identity (T2)	0.01	-0.03	-0.02	0.08
	(0.04)	(0.03)	(0.06)	(0.06)
Ingroup identity (T2)	0.15**	0.19***	-0.41***	0.11
	(0.05)	(0.05)	(0.08)	(0.09)
Dual identity (T2)	0.48***	0.18**	-0.64***	0.44***
	(0.07)	(0.06)	(0.11)	(0.11)
Outgroup identity (T1)	0.01	-0.02	0.07	-0.08
	(0.03)	(0.03)	(0.06)	(0.06)
Ingroup identity (T1)	0.06	0.11**	-0.19*	0.01
	(0.05)	(0.04)	(0.08)	(0.08)
Adjusted R^2^	0.33	0.25	0.37	0.10
F Statistic (5, 350)	36.45***	24.58***	42.70***	8.74***

Note. Standard errors in parentheses.

*p<0.05;

** p<0.01;

***p<0.001

Overall, our dual identity manipulation produced a similar pattern as in Study 2, but it had a weaker impact on attitudes toward non-American Mexicans. Importantly, our hierarchical multivariate regression analyses once again replicated the results of Studies 1 and 2.

#### Mediation analyses

For further support of our hypothesis that the ingroup component of the dual identity is what leads to the positive impact of perceived dual identity on intergroup perceptions despite the presence of the outgroup identity component, we tested the indirect effect of the identity manipulation through perceived ingroup identity on our outcomes. We employed a mediation analysis with the manipulation, setting the dual identity condition as the reference group, perceived ingroup identity as a mediator, and the intergroup orientation variables as dependent variables, using 5000 bootstraps [67] and controlling for perceived ingroup identification in T1. As expected, we found that the indirect effect was significant in all cases for the comparison between the dual-identity condition with the outgroup condition, and that the indirect effect was not significant in any of the cases for comparison with the ingroup condition. In other words, the dual identity manipulation enhanced perceived ingroup identity in a similar manner to the ingroup identity manipulation; perceived ingroup identity, in turn, improved intergroup orientation (see Table 18).

**Table 18:** Mediation analyses of the effect of the identity manipulation on intergroup attitudes through perceived ingroup identification, comparing to the dual identity condition.

Comparison Group	Dependent Variable	Direct Effect	Indirect Effect	Total Effect	Adjusted R^2^
Ingroup condition	Stereotypes	-0.02 [-0.13, 0.08]	-0.03 [-0.07, 0.00]	-0.05 [-0.16, 0.06]	0.32
	Threat	-0.07 [-0.16, 0.02]	-0.04 [-0.08, 0.01]	-0.1* [-0.21, 0.00]	0.42
	Dehumanization	0.03 [-0.06, 0.13]	-0.03 [-0.06, 0.00]	0.01 [-0.09, 0.1]	0.31
	Common identity	-0.1** [-0.17, -0.03]	0.02 [0.00, 0.04]	-0.08* [-0.15, -0.01]	0.24
	General feelings	0.03 [-0.02, 0.09]	0.01 [0.00, 0.04]	0.05 [-0.01, 0.10]	0.25
	Resource allocation	-0.13* [-0.25, -0.01]	0.02 [0.00, 0.04]	-0.12 [-0.23, 0.01]	0.06
	Aggressive policy	-0.09 [-0.20, 0.03]	-0.03 [-0.08, 0.01]	-0.12 [-0.25, 0.01]	0.31
Outgroup condition	Stereotypes	0.04 [-0.08, 0.15]	0.09*** [0.04, 0.14]	0.12* [0.01, 0.24]	0.32
	Threat	-0.06 [-0.18, 0.05]	0.10*** [0.04, 0.16]	0.03 [-0.08, 0.15]	0.42
	Dehumanization	0.01 [-0.09, 0.11]	0.07*** [0.03, 0.12]	0.08 [-0.03, 0.19]	0.31
	Common identity	-0.05 [-0.11, 0.02]	-0.05*** [-0.08, -0.02]	-0.09* [-0.16, -0.02]	0.24
	General feelings	0.06 [0.00, 0.12]	-0.04*** [-0.07, -0.02]	0.02 [-0.05, 0.08]	0.25
	Resource allocation	0.02 [-0.09, 0.14]	-0.05** [-0.09, -0.01]	-0.03 [-0.14, 0.09]	0.06
	Aggressive policy	-0.11 [-0.24, 0.02]	0.10*** [0.04, 0.17]	-0.01 [-0.14, 0.12]	0.31
Control condition	Stereotypes	0.09 [-0.02, 0.2]	0.03 [-0.01, 0.06]	0.12* [0.01, 0.24]	0.32
	Threat	0.00 [-0.09, 0.1]	0.03 [-0.01, 0.07]	0.04 [-0.07, 0.14]	0.42
	Dehumanization	0.05 [-0.05, 0.14]	0.02 [0.00, 0.06]	0.07 [-0.03, 0.17]	0.31
	Common identity	-0.07 [-0.14, 0.01]	-0.02 [-0.04, 0]	-0.08* [-0.16, -0.01]	0.24
	General feelings	-0.02 [-0.08, 0.04]	-0.01 [-0.03, 0.00]	-0.03 [-0.10, 0.03]	0.25
	Resource allocation	-0.02 [-0.14, 0.09]	-0.02 [-0.04, 0.00]	-0.04 [-0.15, 0.08]	0.06
	Aggressive policy	0.00 [-0.12, 0.12]	0.03 [-0.01, 0.07]	0.03 [-0.10, 0.15]	0.31

Note. 95% confidence intervals in brackets.

*p<0.05;

** p<0.01;

***p<0.001

### Discussion

Study 3 generally replicated the patterns from Studies 1 and 2 while offering a number of methodological advantages, including generalizing the effects to an additional intergroup context of Mexican Americans. We found that the experimental manipulation affected both intergroup attitudes towards the dual identity group (Mexican Americans) and towards non-American Mexicans (the gateway group effect). Although the gateway group effects were weaker for Mexicans compared to the Study 2 context of Muslims, the results were still consistent with our predicted pattern.

Replicating Studies 1 and 2, our hierarchical multivariate regression analyses provided evidence consistent with our core theoretical prediction that it is perceived identification that impacts intergroup attitudes. Perceived dual identification was associated with lower levels of the pernicious intergroup outcomes of stereotyping, threat, and dehumanization. Furthermore, perceived dual identification also predicted intergroup perceptions of non-American Muslims. Importantly, all of these regression effects held both when controlling for Time 1 levels of identification.

One reason that our manipulation of perceived identification might have had weaker effects on attitudes towards Mexicans more broadly could be the prevalence of Mexicans in the United States and the proximity between the United States and Mexico. Both variables make direct contact between Americans and Mexicans far more common than contact with Muslims [68]. As a result, there may be less room for the manipulation to affect perceptions of identification for Mexicans compared to Muslims. Furthermore, based on the baseline control condition in Study 2, Americans associate Muslim Americans more with their Muslim (outgroup) identity (M = 81.23, SD = 24.20) than with their American (ingroup) identity (M = 68.95, SD = 31.57, *t*(322) = 4.06, *p* < .001; see Fig 1). On the other hand, based on the baseline perceived identification measure in T1 from Study 3, Americans associate Mexican Americans less with their Mexican identity (M = 69.41, SD = 28.70) than with their American (ingroup) identity (M = 75.23, SD = 27.09, *t*(712) = 2.79, *p* = .005; see Fig 4). As a result, our identity manipulation of dual identity was likely to enhance perceptions of American/ingroup identification for Muslim Americans more than Mexican Americans because Mexican Americans are already seen as identified with the ingroup.

## General discussion

The impact of social identity has been at the center of intergroup psychology research for decades, as numerous studies have yielded robust evidence that the categorization of individuals as part of an ingroup improves attitudes but often creates discriminatory attitudes toward those seen as identifying with one’s outgroup [12; 17]. However, past research has not provided a clear perspective for how people will respond to someone who is identified both with one’s ingroup and an outgroup simultaneously. The intergroup consequences of exposure to dual-identity individuals are both theoretically and practically timely and important. Individuals who explicitly identify with more than one social identity are now represented in the most prominent positions in politics and popular culture, and dual-identity groups are among the fastest-growing demographics [4]. Whereas prior work examined perceptions of gateway groups in terms of identity [e.g., 20], the current work considered the effects of perceived degree of identification. Specifically, our participants exclusively considered groups that hold dual identities (e.g., Muslim American) in order to isolate the effects of variation in the level of perceived identification (e.g., identifying with one’s Muslim and/or American identities) on participants’ attitudes.

In the current research, we examined intergroup attitudes toward dual-identity individuals as well as the corresponding outgroup associated with the dual identity. We hypothesized that the effects of perceiving gateway group members with a dual identity will have effects on intergroup perceptions and evaluations that are similar to perceiving gateway group members primarily identifying with the ingroup. Consistent with this hypothesis, we found that (a) despite the association with an outgroup, when individuals are perceived as holding a dual identity, the attitudes toward them were as positive (i.e., did not significantly differ) as toward others who are perceived to be identified primarily with the ingroup; and (b) when a group is perceived as holding a dual identity, it also improved attitudes toward the outgroup that is associated with the dual identity (i.e. the gateway group effect).

Across three studies in two separate social contexts, we presented both correlational and experimental support for our hypotheses. The reactions of participants to Muslim Americans that were perceived as identified strongly and equally with both their Muslim and American identities were no less positive than the reactions of participants who perceived Muslim Americans as identified only as American. Furthermore, exposure to dual-identified Muslim Americans led participants to report more positive attitudes toward non-American Muslims. Importantly, we replicated these findings in the context of Mexican Americans. This latter finding demonstrates the generalizability of our findings regardless of whether the dual identity consists of two identities on the same dimension (national identity: Mexican American) or two identities on different dimensions (religious and national: Muslim American). In addition, across all studies, we found that perceived dual identity was the main driver of these effects.

The findings of the present research have theoretical and practical implications, with respect to work both on dual identity and on intergroup relations. In particular, our findings that perceiving a group as holding a dual identity has a similar positive impact on intergroup dynamics as seeing them as identified solely with the ingroup contributes to existing theory and research regarding dual identity. Previous research on dual identification has found several beneficial aspects to the subjective experience of holding a dual identity, such as increased wellbeing and creativity [69–71] and decreased intergroup bias [46]. The current research extends this literature from how dual identification affects the self to understanding how perceiving the dual identification of others affects intergroup attitudes and relations. We find that perceiving a group as having a dual identity can positively shape the intergroup attitudes of these observers.

Importantly, many people believe that to be fully accepted by a host culture, minority groups must assimilate with the majority culture [72]. However, our findings suggest that, in terms of intergroup attitudes, explicitly embracing both identities can be similarly beneficial to assimilating to the majority identity. This means that minorities may not need to relinquish any part of their identity as long as they also identify with the majority group identity.

Dual-identified social groups have been marginalized in the context of intergroup relations, subjugated to physical conflict from without and identity conflict from within [73; 74]. In contrast, the current research demonstrates the potential of dual-identified individuals to be harbingers of conflict resolution. This improvement in intergroup attitudes is extended to the corresponding outgroup, with the dual-identity group serving as a gateway to more positive attitudes towards the outgroup. A productive avenue for future research, therefore, would be to explore whether labeling such groups and their members as potential facilitators of conflict resolution can empower them and increase their overall wellbeing while also making them even more effective drivers of improving intergroup relations. Although we have empirically demonstrated the potential of dual-identity groups to facilitate intergroup relations, we want to make clear that it is not the responsibility of such groups to bear the burden of conflict resolution alone. Accordingly, future research might further examine the motivation of dual-identity group members to assume the role of intergroup brokers, and the potential costs that taking on that role might entail.

The present findings extend existing research on the gateway group effect. Previous research has found that mere exposure to a gateway group can improve attitudes toward the relevant outgroup (e.g., Arab citizens of Israel between Israelis and Palestinians, or biracial groups between their monoracial counterparts). The current research, however, illuminates a critical boundary condition of perceived dual identity. In cases where the dual identity group identifies with both identities to the same degree or more strongly with the ingroup, we were able to replicate the gateway group effect. However, when the dual-identity group identifies more strongly with the outgroup, the gateway group effect does not take place and attitudes toward the relevant outgroup may even become more negative. Future research should continue to explore additional boundary conditions for the gateway group effect and to develop social interventions designed to improve intergroup relations.

### Limitations

It is important to note that even though we found robust evidence for the positive impact of explicit dual identification, previous research has clearly found that individuals and groups that hold complex dual identities are most often perceived in a simplistic manner that only focuses on one social identity. For example, cross-categorization research has found that when an individual holds identities that cut across different dimensions (e.g., gender and race), one dimension tends to be the primary focus in the minds of perceivers [24]. Similarly, research on hypodescent has found that when judging mixed-race individuals, perceivers usually focus only on the socially subordinate identity [9; 75]. Accordingly, future research should explore those factors that prevent external perceivers from acknowledging a dual identity when they are exposed to it. For example, research on racial categorization has begun to uncover categorization processes that engage more cognitive effort on behalf of the categorizer [5]. Such processes might lead to reduction in hypodescent and increase the positive impact of biracial identity on inter-racial relations.

In the current work, we focused on the direct effect of perceived dual identity and showed the promise of this experience for improving intergroup relations. However, it is likely that there are multiple variables that may interact with dual identities and lead to different intergroup outcomes [76]. One likely important moderating factor is the context in which exposure to a gateway group occurs. In work on intergroup contact more generally, encounters with members of other groups have a more beneficial impact on intergroup relations when these experiences are associated with successful rather than unsuccessful outcomes [77]. In the example discussed in our introduction, the French-African players were celebrated in the context of a major team victory–winning a World Cup. However, if the French team were to lose, especially in a match against an African team, non-African French perceivers might have a very different response to the dual identity of a gateway group. Consistent with this possibility, Kunst et al. [42] found that when people feel highly vulnerable to injury by an outgroup they may sometimes respond more negatively to members of a group representing a dual (outgroup and ingroup) identity than with only an outgroup identity. Kunst et al. showed that it was uncertainty about the loyalty of dual-identity group members that undermined any benefits of recognizing the ingroup identification of members of such a group. Future research might investigate factors that may moderate responses to groups with dual identities. For instance, it is possible that when people perceive a dual-identity group as one that could make the ingroup particularly vulnerable (e.g., perceiving Mexican Americans as catalysts for undocumented immigration) will lead to more negative responses to the dual identification of groups than we observed in our research.

With respect to testing the boundary conditions of the beneficial impact of exposure to gateway groups on intergroup perceptions and evaluations, we note that Kunst et al. [42] observed the significantly negative impact of others’ dual identity only in contexts (e.g., intergroup relations characterized by threat and vulnerability) in which the loyalty of the dual-identity individual to the ingroup might reasonably be questioned. Under conditions in which loyalty was not in question, the dually identified individual was perceived as positively as an individual who identified only with the ingroup [42]. As a result, in the World Cup example presented earlier, victory may have helped elicit particularly positive perceptions and evaluations of the French-African players (possibly through perceptions of their loyalty and commitment to the team). However, success does not seem to be strictly necessary for the processes we study here: our three studies examined the effect of social identification absent a context of success, and we consistently observed that exposure to gateway group members who identify strongly with the ingroup, even when they also identify strongly with the outgroup, can produce positive intergroup responses. Thus, it would be valuable for future research on the boundary conditions of the impact of exposure to gateway groups to consider neutral as well as positive and negative intergroup contexts.

In addition, within these contexts, other moderating factors might be considered. For example, Kunst et al. [42, Study 4] found that under potentially threatening intergroup conditions, an overt indication of loyalty to the ingroup by a dually-identified individual mitigated the negative response that would otherwise have occurred. Future research might address this issue further by independently, experimentally manipulating intergroup threat along with the extent to which gateway-group members identify with the observer’s ingroup and an outgroup.

Additionally, we found across all three studies that perceived dual identification predicts diminished perceptions of threat, one of the intergroup variables that we consider for perceptions of the dual identity group. Future research might look into whether reduced threat perceptions mediate the link between perceived dual identification and other improved intergroup perceptions. Considering both previous research indicating a moderating role for threat [78] and our findings about how dual-identification of gateway-group members can ameliorate threat also suggests the value of investigating the reciprocal relationships between dual identity, perceived dual identification, and threat perceptions.

While our experimental research (Studies 2 and 3) demonstrated that presenting information about strong identification with the ingroup, either primarily so or as an element of a dual identity that also included strong outgroup identification improved intergroup orientations and typically equivalently so, we caution that this does not necessarily mean that ingroup and dual-identity perceptions of others are necessarily identical in their effects. The common ingroup identity model [46] proposes that these perceptions, while producing many similar benefits, may also have somewhat different dynamics. Thus, future research might also productively consider further investigation of both the common and potentially distinct influences of perceptions on ingroup and dual identity perceptions of gateways groups, compared to outgroup identification perceptions.

Another limitation that we acknowledge is our focus on how majority-group members perceive and evaluate others as a function of how strongly individuals with a dual, majority-minority identify with the different aspects of that identity. It is important to recognize that the ingroup-outgroup dynamics from the perspectives of majority versus minority group-members may differ, and that the perspectives of both groups play a role in shaping intergroup dynamics [79]. Although the current research provides insight into majority group members’ perceptions of gateway groups and the dynamics that shape these perceptions, intergroup relations are influenced as well by the perceptions and responses of minority-group members. Additionally, because intergroup relations reflect the reciprocal actions and reactions of majority- and minority-group members, it is valuable to study the perceptions of both groups in the same context. Thus, future work might pursue a fuller understanding of the role of gateway groups by examining both perspectives, perhaps exploring group status as a moderator of gateway group effects. For example, previous research has found that while majority-group members tend to endorse intergroup assimilation, valuing a single ingroup identity, minority-group members tend to endorse integration, valuing a dual identity [26]. As a result of minority- (versus majority-) group members’ lower preference for a single identity representation and greater preference for a dual identity representation, the gateway group effect may be strengthened for minority-group members. On the other hand, minority-group members may also be more likely to view dually identified individuals as disloyal to their group [2]. In this case, the gateway group effect may be weakened for minority-group members.

## Conclusion

The current set of studies provided the first evidence that reactions toward individuals perceived to have a dual identification produces similarly positive attitudes as perceiving those individuals to be identified with one’s ingroup despite the presence of identification with the outgroup. These studies suggest that individuals and groups with dual identities may play an important role when it comes to improving intergroup relations.

## Supporting information

S1 File As mentioned throughout the text, the supplementary material includes: Information regarding the development of the dual identity measure; The articles used for the identification manipulation; Scale dimensionality; The description of the dual identity groups pilot study. (DOCX)

## References

[1] Young, Z. (2018, July 18). ‘African World Cup winner’ goes down badly with French. The Politico. https://www.politico.eu/article/african-world-cup-winner-goes-down-badly-with-french/

[2] Verkuyten M., Wiley S., Deaux K., & Fleischmann F. (2019). To be both (and more): Immigration and identity multiplicity. *Journal of Social Issues*, 75, 390–413. doi: 10.1111/josi.12324

[3] Levy A., van Zomeren M., Saguy T., & Halperin E. (2017). Intergroup emotions and gateway groups: Introducing multiple social identities into the study of emotions in conflict. *Social and Personality Psychology Compass*, 11, e12320. doi: 10.1111/spc3.12320

[4] Davenport L. D., Iyengar S., & Westwood S. J. (2022). Racial Identity, Group Consciousness, and Attitudes: A Framework for Assessing Multiracial Self‐Classification. *American Journal of Political Science*, 66(3), 570–586. doi: 10.1111/ajps.12674

[5] Pauker K., Meyers C., Sanchez D. T., Gaither S. E., & Young D. M. (2018). A review of multiracial malleability: Identity, categorization, and shifting racial attitudes. *Social and Personality Psychology Compass*, 12, e12392. doi: 10.1111/spc3.12392

[6] Cialdini R. B., Borden R. J., Thorne A., Walker M. R., Freeman S., & Sloan L. R. (1976). Basking in reflected glory: Three (football) field studies. *Journal of Personality and Social Psychology*, 34(3), 366–375. doi: 10.1037/0022-3514.34.3.366

[7] Tajfel H., & Turner J. C. (1979). An integrative theory of intergroup conflict. In Austin W. G. & Worchel S. (Eds.), *The social psychology of intergroup relations* (pp. 33–47). Monterey, CA: Brooks/Cole.

[8] Levy A., Saguy T., van Zomeren M., & Halperin E. (2017). Ingroups, outgroups, and the gateway groups between: The potential of dual identities to improve intergroup relations. *Journal of Experimental Social Psychology*, 70, 260–271. doi: 10.1016/j.jesp.2016.09.011

[9] Ho A. K., Sidanius J., Levin D. T., & Banaji M. R. (2011). Evidence for hypodescent and racial hierarchy in the categorization and perception of biracial individuals. *Journal of personality and social psychology*, 100(3), 492. doi: 10.1037/a0021562 21090902

[10] Verkuyten M. (2010). Assimilation ideology and situational well-being among ethnic minority members. *Journal of Experimental Social Psychology*, 46(2), 269–275. doi: 10.1016/j.jesp.2009.11.007

[11] Brewer M. B. (2017). Intergroup discrimination: Ingroup love or outgroup hate? In Sibley C. G. & Barlow F. K. (Eds.), *The Cambridge handbook of the psychology of prejudice* (pp. 90–110). Cambridge, England: Cambridge University Press. 10.1017/9781316161579.005

[12] Hogg, M. A. (2003). Social identity. In M. R. Leary & J. P. Tangney (Eds.), Handbook of self and identity (p. 462–479). The Guilford Press.

[13] Castano E., Yzerbyt V., & Bourguignon D. (2003). We are one and I like it: The impact of ingroup entitativity on ingroup identification. *European journal of social psychology*, 33(6), 735–754. doi: 10.1002/ejsp.175

[14] Kaiser C. R., & Pratt-Hyatt J. S. (2009). Distributing prejudice unequally: Do Whites direct their prejudice toward strongly identified minorities? *Journal of Personality and Social Psychology*, 96(2), 432–445. doi: 10.1037/a0012877 19159141

[15] Allport G. W. (1954). The nature of prejudice. Addison-Wesley.

[16] Ellemers N., Spears R., & Doosje B. (2002). Self and social identity. Annual Review of Psychology, 53(1), 161–186. doi: 10.1146/annurev.psych.53.100901.135228 11752483

[17] Goldenberg A., Cohen-Chen S., Goyer J. P., Dweck C. S., Gross J. J., & Halperin E. (2018). Testing the impact and durability of a group malleability intervention in the context of the Israeli–Palestinian conflict. *Proceedings of the National Academy of Sciences of the United States of America*, 115, 696–701. doi: 10.1073/pnas.1706800115 29311299 PMC5789904

[18] Levy A., Dovidio J. F. (2021). Intergroup behavior. In Hogg M. (Ed.), *The Oxford research encyclopedia of psychology*. Oxford University Press. 10.1093/acrefore/9780190236557.013.291

[19] Migdal M. J., Hewstone M., & Mullen B. (1998). The effects of crossed categorization on intergroup evaluations: A meta-analysis. British Journal of Social Psychology, 37(3), 303–324. doi: 10.1111/j.2044-8309.1998.tb01174.x

[20] Levy A., Saguy T., Halperin E., & van Zomeren M. (2017). Bridges or barriers? Conceptualization of the role of multiple identity gateway groups in intergroup relations. *Frontiers in Psychology*, 8, 1097. doi: 10.3389/fpsyg.2017.01097 28706501 PMC5489606

[21] Crisp R. J., & Hewstone M. (2007). Multiple social categorization. *Advances in Experimental Social Psychology*, 39, 163–254. doi: 10.1016/S0065-2601(06)39004-1

[22] Kang S. K., & Bodenhausen G. (2015). Multiple identities in social perception and interaction: Challenges and opportunities. *Annual Review of Psychology*, 66, 547–574. doi: 10.1146/annurev-psych-010814-015025 25061671

[23] Chen J. M., & Hamilton D. L. (2012). Natural ambiguities: Racial categorization of multiracial individuals. *Journal of Experimental Social Psychology*, 48(1), 152–164. doi: 10.1016/j.jesp.2011.10.005

[24] Hagendoorn L., & Henke R. (1991). The effect of multiple category membership on intergroup evaluations in a north Indian context: Class, caste and religion. *British Journal of Social Psychology*, 30(3), 247–260. doi: 10.1111/j.2044-8309.1991.tb00942.x

[25] Chen J. M., Moons W. G., Gaither S. E., Hamilton D. L., & Sherman J. W. (2014). Motivation to control prejudice predicts categorization of multiracials. *Personality and Social Psychology Bulletin*, 40, 590–603. doi: 10.1177/0146167213520457 24458216

[26] Dovidio, J. F., Gaertner, S. L., Schnabel, N., Saguy, T., & Johnson, J. (2010). Recategorization and prosocial behavior. *The Psychology of Prosocial Behavior*: *Group Processes*, *Intergroup Relations*, *and Helping*, 289–309.

[27] Gómez Á., Dovidio J. F., Gaertner S. L., Fernández S., & Vázquez A. (2013). Responses to endorsement of commonality by ingroup and outgroup members: the roles of group representation and threat. *Personality & Social Psychology Bulletin*, 39, 419–431. doi: 10.1177/0146167213475366 23456560

[28] Wildschut T., & Insko C. A. (2007). Explanations of interindividual–intergroup discontinuity: A review of the evidence. European Review of Social Psychology, 18(1), 175–211. doi: 10.1080/10463280701676543

[29] Halabi, S., & Nadler, A. (2017). The intergroup status as helping relations model: Giving, seeking and receiving help as tools to maintain or challenge social inequality. In E. van Leeuwen & H. Zagefka (Eds.), *Intergroup helping* (pp. 205–221). Springer International Publishing. 10.1007/978-3-319-53026-0_10

[30] Dunham Y. (2011). An angry = Outgroup effect. *Journal of Experimental Social Psychology*, 47(3), 668–671. doi: 10.1016/j.jesp.2011.01.003

[31] Pratto F., & John O. P. (1991). Automatic vigilance: The attention-grabbing power of negative social information. *Journal of Personality and Social Psychology*, 61(3), 380–391. doi: 10.1037//0022-3514.61.3.380 1941510

[32] Fiske S. T. (1980). Attention and weight in person perception: The impact of negative and extreme behavior. *Journal of Personality and Social Psychology*, 38(6), 889–906. doi: 10.1037/0022-3514.38.6.889

[33] Rozin P., & Royzman E. B. (2001). Negativity bias, negativity dominance, and contagion. *Personality and Social Psychology Review*, 5(4), 296–320. doi: 10.1207/S15327957PSPR0504_2

[34] Urada D, Stenstrom DM, Miller N. (2007) Crossed categorization beyond the two-group model. *Journal of Personality and Social Psychology*, 92(4), 649–664. doi: doi: 10.1037/0022-3514.92.4.649 17469950

[35] Yzerbyt V. Y., Leyens J. P., & Bellour F. (1995). The ingroup overexclusion effect: Identity concerns in decisions about group membership. *European Journal of Social Psychology*, 25(1), 1–16. doi: 10.1002/ejsp.2420250102

[36] Rodeheffer C. D., Hill S. E., Lord C. G. (2012). Does this recession make me look Black? The effect of resource scarcity on the categorization of multiracial faces. *Psychological Science*, 23, 1476–1478. doi: 10.1177/0956797612450892 23085641

[37] Wilton L. S., Rattan A., & Sanchez D. T. (2018). White’s perceptions of biracial individuals’ race shift when biracials speak out against bias. *Social Psychological and Personality Science*, 9, 953–961. doi: 10.1177/1948550617731497

[38] Gaertner S. L., Dovidio J. F., Banker B. S., Houlette M., Johnson K. M., & McGlynn E. A. (2000). Reducing intergroup conflict: From superordinate goals to decategorization, recategorization, and mutual differentiation. *Group Dynamics*: *Theory*, *Research*, *and Practice*, 4 *(*1), 98–114. doi: 10.1037/1089-2699.4.1.98

[39] Brewer M. B. (1999). The psychology of prejudice: Ingroup love and outgroup hate?. *Journal of social issues*, 55(3), 429–444. doi: 10.1111/0022-4537.00126

[40] Halevy N., Bornstein G., & Sagiv L. (2008). "In-group love" and "out-group hate" as motives for individual participation in intergroup conflict: A new game paradigm. *Psychological Science*, 19(4), 405–411. doi: 10.1111/j.1467-9280.2008.02100.x 18399895

[41] Waytz A., Young L. L., & Ginges J. (2014). Motive attribution asymmetry for love vs. hate drives intractable conflict. Proceedings of the National Academy of Sciences, 111(44), 15687–15692. doi: 10.1073/pnas.1414146111 25331879 PMC4226129

[42] Kunst J. R., Thomsen L., & Dovidio J. F. (2019). Divided loyalties: Perceptions of disloyalty underpin bias toward dually-identified minority-group members. *Journal of Personality and Social Psychology*, 117, 807–838. doi: 10.1037/pspi0000168 30382739

[43] Levy A., Zezelj I., Branković M., Dusanic S., van Zomeren M., Saguy T., et al. (2019). Complex social identities and intergroup relations: Gateway groups in the Western Balkans. *Social Psychology*, 50, 201–206. doi: 10.1027/1864-9335/a000379

[44] Levy A., Halperin E., van Zomeren M., & Saguy T. (2018). Inter-racial gateways: The potential of biracials to reduce threat and prejudice in inter-racial dynamics. *Race and Social Problems*, 11, 119–132. doi: 10.1007/s12552-018-9257-x

[45] Love A., & Levy A. (2019). Bridging group divides: A theoretical overview of the “what” and “how” of gateway groups. *Journal of Social Issues*, 75, 414–435. doi: 10.1111/josi.12327

[46] Gaertner S. L., Dovidio J. F., Guerra R., Hehman E., & Saguy T. (2016). A common ingroup identity: A categorization-based approach for reducing intergroup bias. In Nelson T. (Ed.), *Handbook of prejudice*, *discrimination*, *and stereotyping* (2nd ed., pp. 433–454). New York, NY: Psychology Press.

[47] Brown R., & Hewstone M. (2005). An integrative theory of intergroup contact. *Advances in Experimental Social Psychology*, 37, 255–343. doi: 10.1016/S0065-2601(05)37005-5

[48] Fiske, S. T., & Taylor, S. E. (2017). *Social cognition* (3rd ed.). Mcgraw-Hill Book Company.

[49] Mackie, D. M., & Smith, E. R. (2015). Intergroup emotions. In M. Mikulincer, P. R. Shaver, J. F. Dovidio, J. F., & J. A. Simpson (Eds.), *APA handbook of personality and social psychology*, *Volume 2*: *Group and intergroup processes* (pp. 263–293). Washington, DC: American Psychological Association.

[50] Kteily N., Bruneau E., Waytz A., & Cotterill S. (2015). The ascent of man: Theoretical and empirical evidence for blatant dehumanization. *Journal of Personality and Social Psychology*, 109(5), 901–931. doi: 10.1037/pspp0000048 26121523

[51] Sinclair S., Sidanius J., & Levin S. (1998). The interface between ethnic and social system attachment: The differential effects of hierarchy-enhancing and hierarchy-attenuating environments. *Journal of Social Issues*, 54, 741–757. doi: 10.1111/j.1540-4560.1998.tb01246.x

[52] Gaertner S. L., & Dovidio J. F. (2000). *Reducing intergroup bias*: *The common ingroup identity model*. Philadelphia, PA: The Psychology Press.

[53] R Core Team. (2020). *R*: *A language and environment for statistical computing*. R Foundation for Statistical Computing. https://www.R-project.org/

[54] Lewis, B. (1993). *Islam and the West*. OUP USA.

[55] Said, E. W. (2012). *Culture and imperialism*. Vintage.

[56] Simon B., Reichert F., & Grabow O. (2013). When dual identity becomes a liability: Identity and political radicalism among migrants. *Psychological Science*, 24, 251–257. doi: 10.1177/0956797612450889 23319402

[57] Faul F., Erdfelder E., Lang A.-G., & Buchner A. (2009). Statistical power analyses using G*Power 3.1: Tests for correlation and regression analyses. *Behavior Research Methods*, 41, 1149–1160. doi: 10.3758/BRM.41.4.1149 19897823

[58] Thompson M. M., Zanna M. P., & Griffin D. W. (1995). Let’s not be indifferent about (attitudinal) ambivalence. *Attitude strength*: *Antecedents and consequences*, 4, 361–386.

[59] Vaes J., Latrofa M., Suitner C., & Arcuri L. (2019). They are all armed and dangerous! Biased language use in crime news with ingroup and outgroup perpetrators. *Journal of Media Psychology*: *Theories*, *Methods*, *and Applications*, 31(1), 12.

[60] Stephan W. S., Ybarra O., & Rios K. (2016). Intergroup threat theory. In Nelson T.(Ed.), *Handbook of prejudice*, *discrimination*, *and stereotyping* (2nd ed., pp. 33–56). New York, NY: Psychology Press.

[61] Leidner B., Castano E., & Ginges J. (2013). Dehumanization, retributive and restorative justice, and aggressive versus diplomatic intergroup conflict resolution strategies. *Personality and Social Psychology Bulletin*, 39(2), 181–192. doi: 10.1177/0146167212472208 23386655

[62] Luguri J. B., Napier J. L., & Dovidio J. F. (2012). Reconstruing intolerance: Abstract thinking reduces conservatives’ prejudice against nonnormative groups. *Psychological Science*, 23, 756–763. doi: 10.1177/0956797611433877 22653799

[63] Kahneman D., Knetsch J. L., & Thaler R. H. (1986). Fairness and the assumptions of economics. *Journal of Business*, 59, 285–300.

[64] O’Brien R. G., & Kaiser M. K. (1985). MANOVA method for analyzing repeated measures designs: an extensive primer. *Psychological Bulletin*, 97(2), 316. 3983301

[65] Osborne J. W., & Waters E. (2002). Four assumptions of multiple regression that researchers should always test. *Practical Assessment*, *Research*, *and Evaluation*, 8(1), 2.

[66] Tabachnick B. G., & Fidell L. S. (2001). *Using Multivariate Statistics* (4th ed.). Needham Heights, MA: Allyn and Bacon

[67] Tingley D., Yamamoto T., Hirose K., Imai K., & Keele L. (2014). mediation: R package for causal mediation analysis. *Journal of Statistical Software*, Vol. 59(5), 1–38.

[68] González R., & Brown R. (2006). Dual identities in intergroup contact: Group status and size moderate the generalization of positive attitude change. *Journal of Experimental Social Psychology*. doi: 10.1016/j.jesp.2005.11.008

[69] Jetten J., Haslam C., Haslam S. A., Dingle G., & Jones J. M. (2014). How groups affect our health and well-being: The path from theory to policy. *Social Issues and Policy Review*, 8, 103–130. doi: 10.1111/sipr.12003

[70] Steffens N. K., Gocłowska M. A., Cruwys T., & Galinsky A. D. (2016). How multiple social identities are related to creativity. *Personality and Social Psychology Bulletin*, 42, 188–203. doi: 10.1177/0146167215619875 26646430

[71] Repke L., & Benet-Martínez V. (2018). The (diverse) company you keep: Content and structure of immigrants’ social networks as a window into intercultural relations in Catalonia. *Journal of Cross-Cultural Psychology*, 49, 924–944. doi: 10.1177/0022022117733475

[72] Politi E., Roblain A., Gale J., Licata L., & Staerklé C. (2020). If you want to be one of us, then become like us: The evaluation of naturalization applicants by host nationals. *European Journal of Social Psychology*, 50(4), 839–856. doi: 10.1002/ejsp.2663

[73] Gaither S. E., Sommers S. R., & Ambady N. (2013). When the half affects the whole: Priming identity for biracial individuals in social interactions. *Journal of Experimental Social Psychology*, 49, 368–371. doi: 10.1016/j.jesp.2012.12.012

[74] Verkuyten M., & Reijerse A. (2008). Intergroup structure and identity management among ethnic minority and majority groups: The interactive effects of perceived stability, legitimacy, and permeability. *European Journal of Social Psychology*, 38, 106–127. doi: 10.1002/ejsp.395

[75] Peery D., & Bodenhausen G. V. (2008). Black+ White = Black: Hypodescent in reflexive categorization of racially ambiguous faces. *Psychological Science*, 19(10), 973–977. doi: 10.1111/j.1467-9280.2008.02185.x 19000204

[76] Benet-Martínez V., Leu J., Lee F., & Morris M. W. (2002). Negotiating biculturalism: Cultural frame switching in biculturals with oppositional versus compatible cultural identities. *Journal of Cross-Cultural* Psychology, 33, 492–516. doi: 10.1177/0022022102033005005

[77] Blanchard F. A., Adelman L., & Cook S. W. (1975). Effect of group success and failure upon interpersonal attraction in cooperating interracial groups. *Journal of Personality and Social Psychology*, 31(6), 1020–1030.

[78] Urbiola A., Willis G. B., Ruiz‐Romero J., & Moya M. (2018). Does a multicultural perspective shape unbiased minds? The moderating role of outgroup threat. Journal of Applied Social Psychology, 48(11), 608–617. doi: 10.1111/jasp.12551

[79] Lammers J., Galinsky A. D., Gordijn E. H., & Otten S. (2012). Power increases social distance. *Social Psychological and Personality Science*. doi: 10.1177/1948550611418679

