# Supplementary material for: Ingroup love, outgroup hate, and the gateway group effect: Comparing the direct and indirect impact of dual versus single identification
Source: PLoS One. 2023 Aug 16;18(8):e0287631. doi: 10.1371/journal.pone.0287631 (PMC10431672; doi:10.1371/journal.pone.0287631)
Supplement: S1 File — (DOCX) [file pone.0287631.s001.docx]

SUPPLEMENTARY MATERIALS

**Ingroup Love, Outgroup Hate, and the Gateway Group Effect:**

**Comparing the Direct and Indirect Impact of Dual versus Single Identification**

By

Aharon Levy, Adam Galinsky, Christine Q. Nguyen, Tamar Saguy, Elif G. Ikizer, and John F. Dovidio

*Table Contents*

| Section | Page |
| --- | --- |
|  |  |
| Development of dual identity measure | 3 |
| Manipulation articles | 10 |
| Scale dimensionality | 16 |
| Dual identity groups pilot study | 17 |

**Development of dual identity measure: Identifying with Both Identities to a High *and* Similar Degree**

Because current measures of dual identification do not allow us to test our core predictions, we created a new measure for it. Conceptually, a dual identity is derived from three elements: (a) the identification with one identity (e.g., home country), (b) the identification with another identity (e.g. host country), and (c) the relative strength of the two identities, that is, whether they are similar or one is stronger than the other. The premise of our approach to measuring dual identity is that high dual identification requires one to identify with both identities to a high *and* similar degree. Even if both identities are high but one of them is substantially more significant than the other, this diminishes the dual nature of the identification. This is analogous to colors. For example, purple is a combination of two other colors, red and blue. Creating the color purple requires not only both red and blue but also a similar amount of both colors because an imbalance in one of the colors will lead to either a colder shade of red or a warmer shade of blue but not purple.

Despite the importance of all three elements—identification with one identity, identification with another identity, and the relative strength of the two identities—most existing measures do not take all three elements into account. For example, in understanding the dual identity of Turkish immigrants in Germany, Simon, Reichert, and Grabow (2013) asked whether participants felt they “belong to both the Turks and the Germans” or “have many similarities with Germans as well as Turks.” While such items can give an indication of duality, they do not give an indication of each identity separately and thus can’t distinguish between someone who has a clear disparity between the two identities from someone who does not. Other existing dual identity measures treat identification as a binary construct (dual identity: yes or no), or a categorical construct (e.g., assimilation, integration, separation, or marginalization), and thus are not sensitive to variation on a continuous scale (Baysu, Phalet, & Brown, 2011; Berry, 1977; Crawford, Allison, Zamboni, & Soto, 2002).

These examples highlight that an effective measure of dual identification requires measuring both identities separately and on continuous scales. Take for example two people, one who identifies with two separate identities to a similar degree, a 6 on a 10-point scale versus another who identifies with both identities at a 9. Or a person who identifies with one identity moderately (6) and the other identity lowly (3) versus someone with the same disparity but higher overall indentation of 9 and 6. Even though such distinctions are obviously significant, only a measure that takes into account all three elements will be sensitive to them.

In addition, almost all past measures focus on the subjective experience of identification and not on external perception (i.e., designed only for the dual identity holders but not for people who interact with them). This focus on the identity holder’s subjective experience doesn't allow researchers to distinguish between an ingroup vs. an outgroup identity because for each of these identities are ingroup for group member.

To create a measure of dual identification that meets the above criteria, we adapted the logic from the acculturation literature which allows for both main effects and complex interactions between the two identities (Benet-Martínez, Leu, Lee, & Morris, 2002; Tadmor, Galinsky, & Maddux, 2012; Ward & Kennedy, 1994). We also modeled our approach on previous work on mixed emotions (Moore & Martin, 2022) and more specifically on attitude ambivalence (see Kaplan, 1972; Thompson, Zanna, & Griffin, 1995).

Whereas much of the traditional research conceived of attitudes as unidimensional evaluative summary statements (Fazio, 1986), various lines of research conceptualized attitudinal ambivalence as a form of conflict experienced as the result of having inconsistent, and potentially contradictory, beliefs or feelings about an attitudinal object (e.g., a social group). Research on race relations, for example, demonstrated that greater attitudinal ambivalence of White American participants toward Black Americans, operationalized as the mathematical product of Pro-Black and Anti-Black sentiments, predicted greater amplification of responses to Black individuals (Hass, Katz, Rizzo, Bailey, & Eisenstadt, 1991). Although our focus was not on assessing attitudinal ambivalence and its consequences, the new measure that we constructed drew on the general principle in the ambivalence literature that examining two constituent elements in combination has the potential to offer unique insights into the psychology of intergroup relations.

Our measure not only allowed us to capture perceived identification with an ingroup identity separately from perceived identification with the outgroup identity, but it also allowed us to capture the relative strength of the two identities, for example, whether they are similar versus one is stronger is than the other. Our simple and straightforward measure of dual identity accounts for both identities as well as their relative strengths by measuring each identification on a continuous scale. Furthermore, it allows researchers to measure dual identification from an external perspective as well.

Accordingly, to measure dual identification, we developed a new measure and formula. We first considered the applicability of a new dual identity measure, modeled after either the research of Hass et al. (1991), representing dual identity as the product of the strength of two constituent identities, or the work of Kaplan (1972), reflecting the sum of the constituent components to distinguish indifference (a small sum) from ambivalence (a large sum). However, we concluded that neither of these captured a key conceptual element of a strong dual identity – little disparity between the strength of the two constituent identities.

Based on the work done in the realm of measuring ambivalence (Thompson, Zanna, & Griffin, 1995), we experimented with different mathematical formulas to account for both the degree and disparity of the two identities in a single score. Because an effective dual identity measure requires a) high identification with both identities and b) little disparity between the two identities, we devised the dual identity score (*DI*) to capture the combination of the average score of both identities (identity one: *ID_1_* and identity two: *ID_2_*) and the absolute value of the delta between them.

***DI* = (*ID_1_ + ID_2_)/2-ABS(ID_1_ – ID_2_)***

This equation was designed to produce high dual identity scores when both identities are high and there is little disparity between them. The equation corresponds exactly to the formula devised by Thompson, Zanna, and Griffin (1995) for measuring attitudinal ambivalence. However, whereas the two constituent values used by Thompson et al. were the strength of an individual’s positive and negative evaluations of an attitude object. In our application, the two values represent the strength that participants perceived others—specifically, members of a gateway group—to hold two different group identities.

Table 1 highlights how the value of the dual identity formula rises when either the level of identification with both identities grows or the disparity between the two identities diminishes, but the score rises the most when both these conditions are satisfied. Consider an individual with two identities both at a level of 6/10 versus an individual with one 8/10 identity and one 4/10 identity. If we were to simply take the sum of the two identities, the dual identity level for both individuals would be the same (12). However, as can be seen in Table 1, due to the fact that our formula accounts for the disparity between identities, it allows us to distinguish between each of these individuals: the first individual (both identities are scored a 6) receives a score of 6 and the second individual (the identities are scored 4 and 8) receives a score of 2.

***Table 1.*** *Iterations of possible dual identity scores based on our new dual identity formula. Each identity is scored using scales from 1 to 10. Our dual identity score (DI) captures the combination of the average score of both identities (identity one: ID1 and identity two: ID2) and the absolute value of the delta between them. DI =* ***(ID1 + ID2)/2-ABS(ID1 – ID2)***

|  |  | **Identity A** | | | | | | | | | |
| --- | --- | --- | --- | --- | --- | --- | --- | --- | --- | --- | --- |
|  |  | 1 | 2 | 3 | 4 | 5 | 6 | 7 | 8 | 9 | 10 |
| **Identity B** | 1 | **1** | 0.5 | 0 | -0.5 | -1 | -1.5 | -2 | -2.5 | -3 | -3.5 |
|  | 2 | 0.5 | **2** | 1.5 | 1 | 0.5 | 0 | -0.5 | -1 | -1.5 | -2 |
|  | 3 | 0 | 1.5 | **3** | 2.5 | 2 | 1.5 | 1 | 0.5 | 0 | -0.5 |
|  | 4 | -0.5 | 1 | 2.5 | **4** | 3.5 | 3 | 2.5 | 2 | 1.5 | 1 |
|  | 5 | -1 | 0.5 | 2 | 3.5 | **5** | 4.5 | 4 | 3.5 | 3 | 2.5 |
|  | 6 | -1.5 | 0 | 1.5 | 3 | 4.5 | **6** | 5.5 | 5 | 4.5 | 4 |
|  | 7 | -2 | -0.5 | 1 | 2.5 | 4 | 5.5 | **7** | 6.5 | 6 | 5.5 |
|  | 8 | -2.5 | -1 | 0.5 | 2 | 3.5 | 5 | 6.5 | **8** | 7.5 | 7 |
|  | 9 | -3 | -1.5 | 0 | 1.5 | 3 | 4.5 | 6 | 7.5 | **9** | 8.5 |
|  | 10 | -3.5 | -2 | -0.5 | 1 | 2.5 | 4 | 5.5 | 7 | 8.5 | **10** |

Because dual identity represents a composite of different factors that can change independently from one another, conceptually there can be different combinations that can produce a similar score. For example, high identification with high disparity is similar to lower identification with lower disparity. As can be seen in Table 1, the new formula scores such different and equivalent combinations in a similar manner (e.g., the score of 1 for the combinations of 4:2, 7:3, 10:4).

In the following tables we proved details of other potential variations of the formula that yielded less accurate scores.

| ***Option 1.*** *Multiplied identities.*  *As can be seen in this table, this formula only accounts for the level of identification and does not account for the relative strength. As a result there are scores with disparate dual identity strength that are higher than scores with equal dual identity strength.* | ***Option 2.*** *Multiplied identities divided by delta.*  *In order to avoid dividing by zero, a 1 must be added to the delta score. This leads to no variance when one of the scores equals 1.* |
| --- | --- |
| \|  \|  \| ***ID1*ID2*** \| \| \| \| \| \| --- \| --- \| --- \| --- \| --- \| --- \| --- \| \|  \|  \| Ingroup Identity \| \| \| \| \| \| Outgroup Identity \|  \| 1 \| 2 \| 3 \| 4 \| 5 \| \| 1 \| **1.0** \| 2.0 \| 3.0 \| 4.0 \| 5.0 \| \| 2 \| 2.0 \| **4.0** \| 6.0 \| 8.0 \| 10.0 \| \| 3 \| 3.0 \| 6.0 \| **9.0** \| 12.0 \| 15.0 \| \| 4 \| 4.0 \| 8.0 \| 12.0 \| **16.0** \| 20.0 \| \| 5 \| 5.0 \| 10.0 \| 15.0 \| 20.0 \| **25.0** \| | \|  \|  \| ***(ID1*ID2)/(ABS(ID1-ID2)+1)*** \| \| \| \| \| \| --- \| --- \| --- \| --- \| --- \| --- \| --- \| \|  \|  \| Ingroup Identity \| \| \| \| \| \| Outgroup Identity \|  \| 1 \| 2 \| 3 \| 4 \| 5 \| \| 1 \| **1.0** \| 1.0 \| 1.0 \| 1.0 \| 1.0 \| \| 2 \| 1.0 \| **4.0** \| 3.0 \| 2.7 \| 2.5 \| \| 3 \| 1.0 \| 3.0 \| **9.0** \| 6.0 \| 5.0 \| \| 4 \| 1.0 \| 2.7 \| 6.0 \| **16.0** \| 10.0 \| \| 5 \| 1.0 \| 2.5 \| 5.0 \| 10.0 \| **25.0** \| |
| ***Option 3.*** *Sum of identities minus delta.*  *As can be seen in this table, the result of this formula is simply 2X the smaller value. This leads to very little variance in the scores.* | ***Option 4.*** *Average of identities minus delta.*  *The selected formula. This formula provides a continuous score that accounts for both combined and relative identity strength.* |
| \|  \|  \| ***(ID1+ID2)-ABS(ID1-ID2)*** \| \| \| \| \| \| --- \| --- \| --- \| --- \| --- \| --- \| --- \| \|  \|  \| Ingroup Identity \| \| \| \| \| \| Outgroup Identity \|  \| 1 \| 2 \| 3 \| 4 \| 5 \| \| 1 \| **2** \| 2 \| 2 \| 2 \| 2 \| \| 2 \| 2 \| **4** \| 4 \| 4 \| 4 \| \| 3 \| 2 \| 4 \| **6** \| 6 \| 6 \| \| 4 \| 2 \| 4 \| 6 \| **8** \| 8 \| \| 5 \| 4 \| 4 \| 6 \| 8 \| **10** \| | \|  \|  \| ***(ID1+ID2)/2-ABS(ID1-ID2)*** \| \| \| \| \| \| --- \| --- \| --- \| --- \| --- \| --- \| --- \| \|  \|  \| Ingroup Identity \| \| \| \| \| \| \| Outgroup Identity \|  \| 1 \| 2 \| 3 \| 4 \| 5 \| \| \| 1 \| **1** \| 0.5 \| 0 \| -0.5 \| -1 \| \| \| 2 \| 0.5 \| **2** \| 1.5 \| 1 \| 0.5 \| \| \| 3 \| 0 \| 1.5 \| **3** \| 2.5 \| 2 \| \| \| 4 \| -0.5 \| 1 \| 2.5 \| **4** \| 3.5 \| \| \| 5 \| -1 \| 0.5 \| 2 \| 3.5 \| **5** \| \| |

We explored the results associated with this new measure of dual identity in each of our three studies. While the new measure is indirect in that participants are not asked explicitly about their experience of a dual identity, which could limit the degree to which it might be affected by strategic responses (e.g., social desirability or demand characteristic influence), empirically it was highly related to the direct measure of dual identity used by Simon et al. (2013). In Study 1, for example, the two were strongly correlated, r(142) = 0.74, p < .001. The new measure also did not account for meaningful additional variance for our outcomes of interest beyond the Simon et al. (2013) measure across our studies. Thus, we report the results for the previously validated Simon et al. (2013) measure in the analyses we report in the main text.

**Manipulation Articles**

The independent variable in this study was the Muslim American gateway group's form of identification. There were four conditions represented by the content of a news article that participants were asked to read as “An article that was recently published about a survey conducted among Muslim Americans that dealt with the issue of the identity of this population in the USA.” The articles were framed as appearing on CBS news which was chosen due to its relatively neutral political affiliation.

Three of the conditions systematically presented information about how Muslim Americans socially identified; the fourth condition was a control condition in which participants read about an unrelated topic. In each of these three Muslim American identity conditions, the article that participants read described the results of a survey of “more than 1000 Muslims ages 18 and up all over the country,” representing “one of the most thorough surveys on this matter to date.” These three conditions differed in the headline and the purported results.

In the Predominant American Identity condition, the headline of an ostensible news article read, “Survey: Majority of Muslim Americans identify mostly with their American identity,” and the text reported that “of the Muslim American participants, 83% claimed that being American plays the most significant role in their personal identity. Hence, the vast majority of Muslim Americans see themselves as primarily American.” The text elaborated on these findings and including corresponding quotes and commentary. (See below for the complete texts.)

In the Predominant Muslim Identity Condition, the headline indicated, “Survey: Majority of Muslim Americans identify with their Muslim identity,” and reported, in parallel fashion, that “83% claimed that being Muslim plays the most significant role in their personal identity” that “Muslim Americans see themselves primarily as Muslim” and with other text appropriately modified.

In the Dual Identity condition, the headline stated, “Majority of Muslim Americans identify with both of their identities to the same degree.” This article explained that “83% claimed that the USA plays a significant role in their personal identity together with their Muslim identity. Hence, the vast majority of Muslim Americans see themselves as American and at the same time identify as part of the Muslim world.” The text elaborating on these findings was modified in a way that paralleled the other two identity conditions. The article in the fourth condition, the control condition, was of comparable length and format but was about astronomy, a topic not related to the gateway group in any way.

All four articles are provided below.

***Ingroup identity article:***


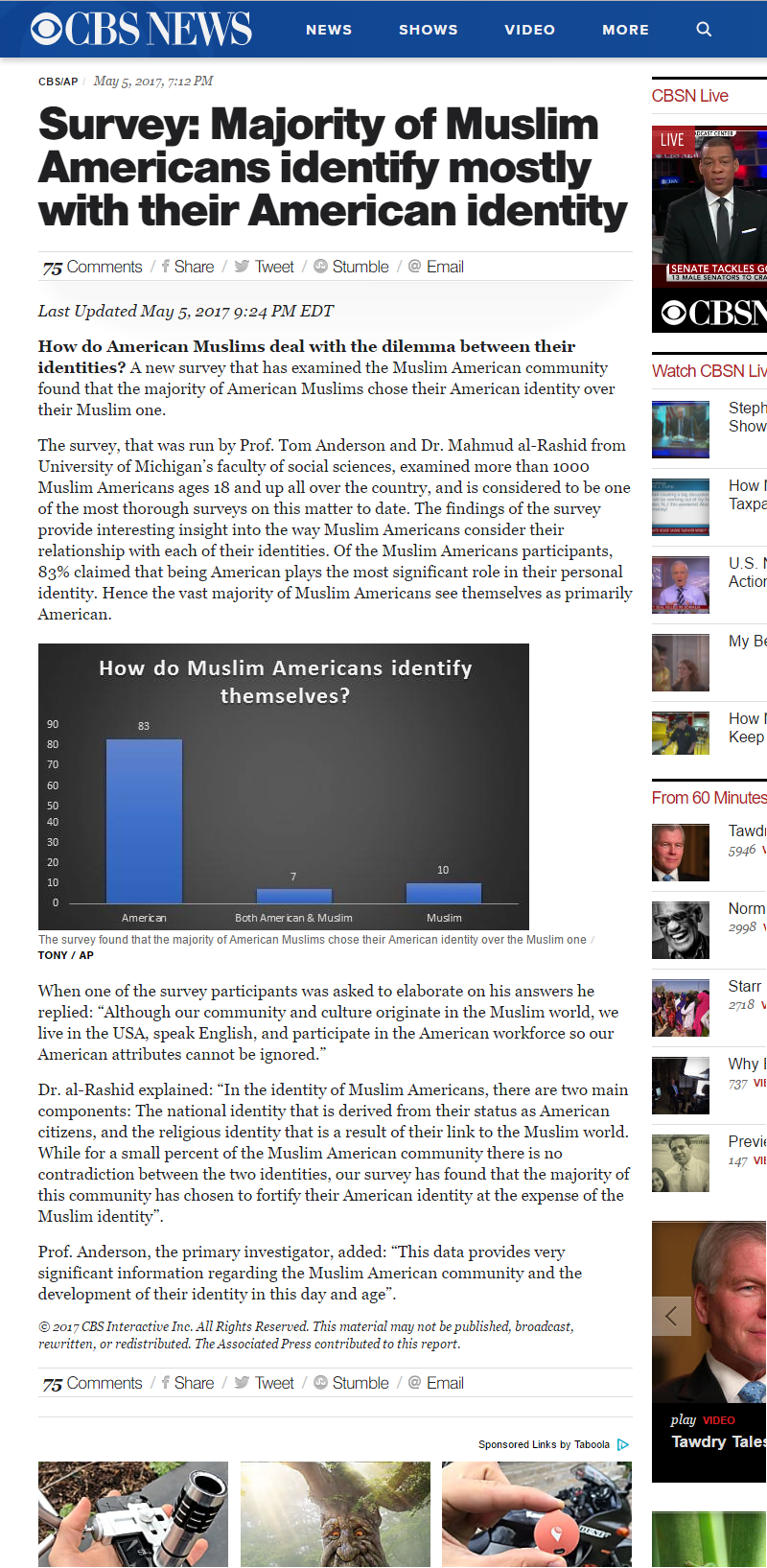


***Outgroup identity article:***


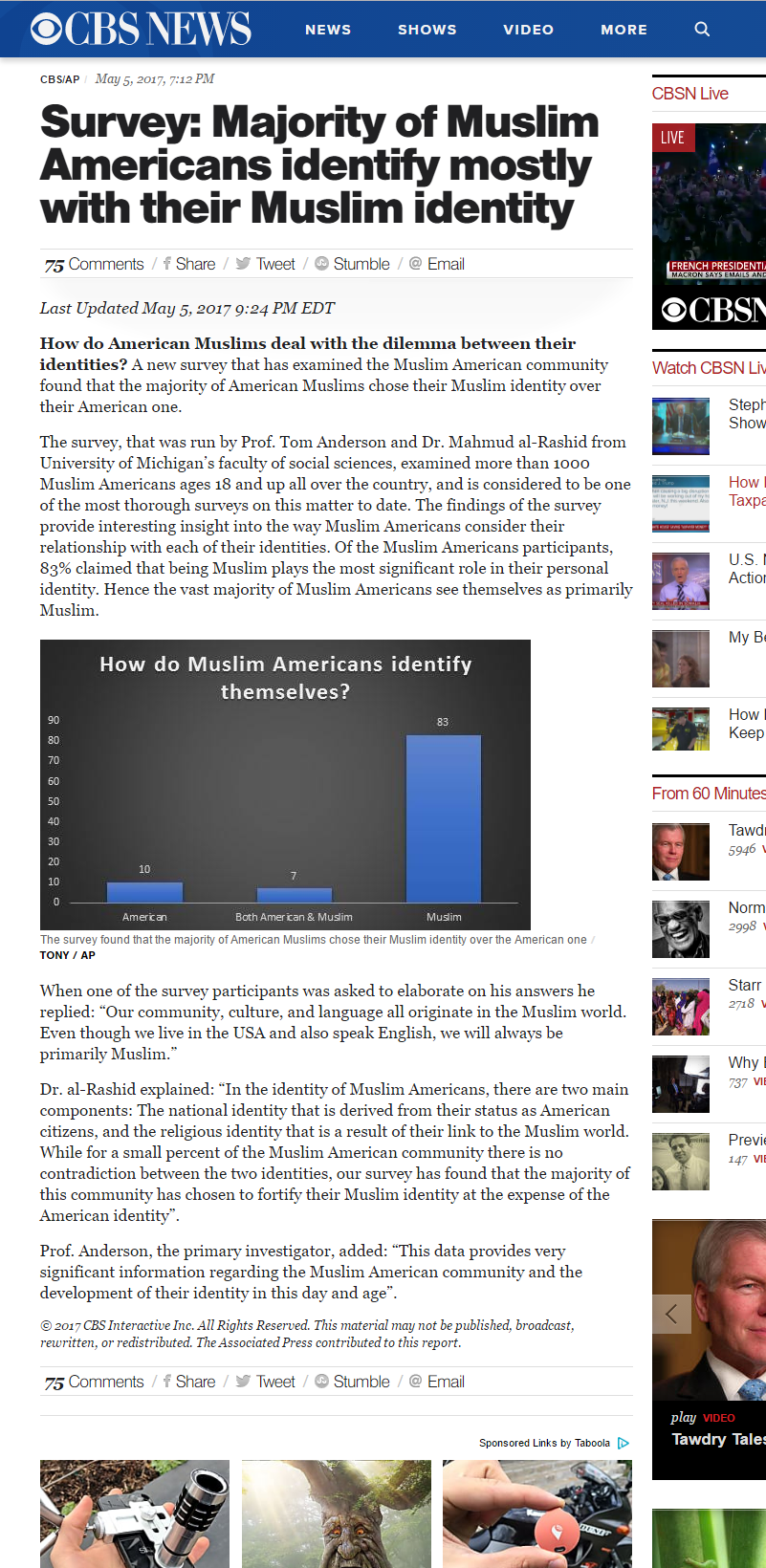


***Dual identity article:***


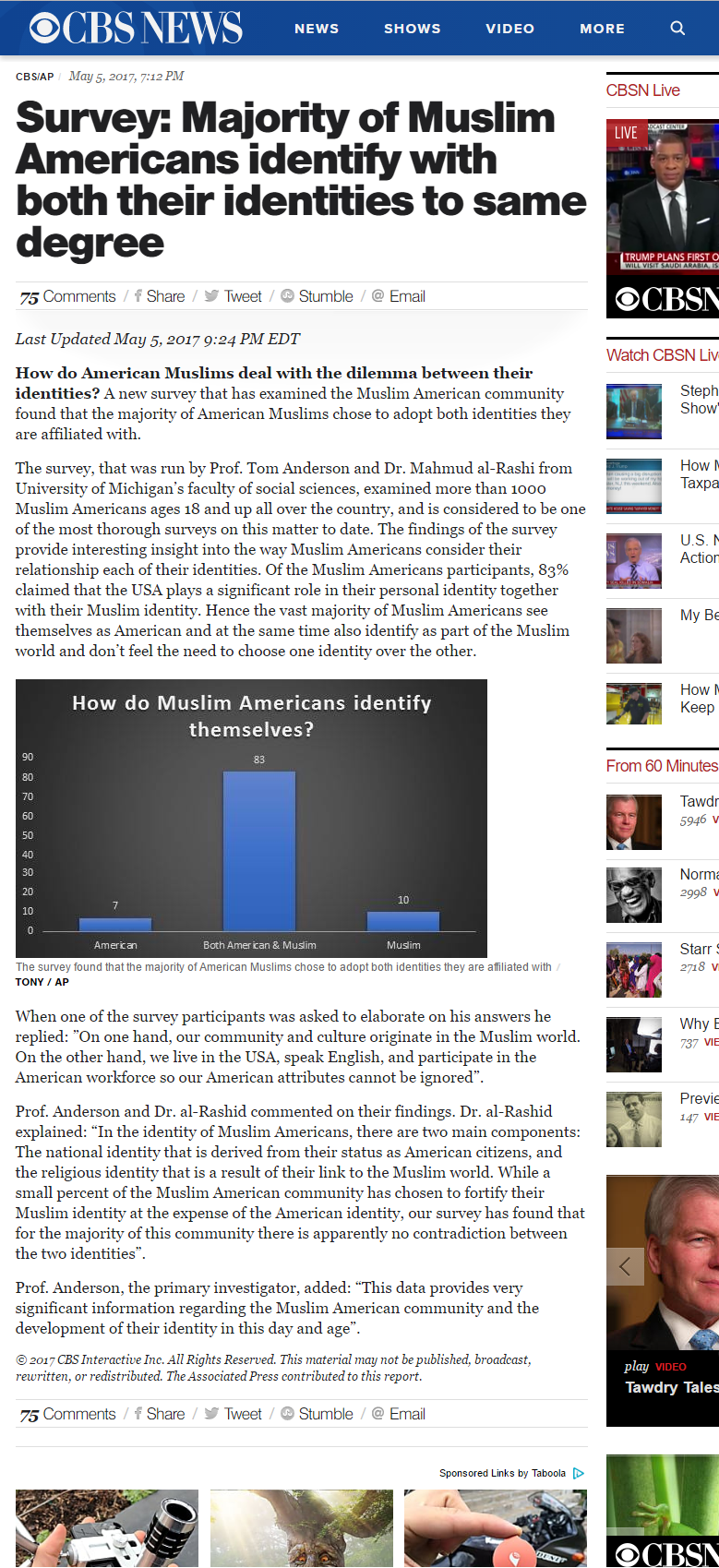


***Control condition article:***


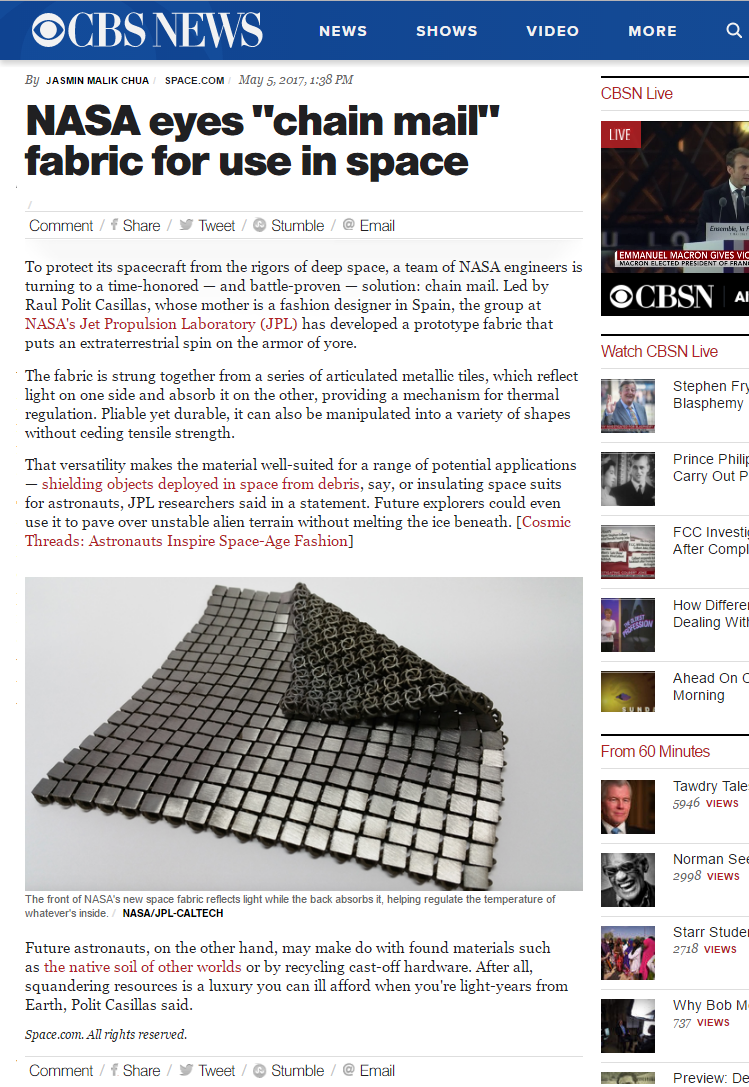


# Scale dimensionality

Parallel analyses using the psych package in R suggest that, for the majority of our measures in all three studies, the measure’s items load onto one factor and onto one component.

*Perceived dual identity**.* As noted in the main text, the original measure by Simon et al. (2013) includes four items. One of the items, however, led to notably low internal consistency (α_Study 1_ = 0.62; α_Study 2_ = 0.70; α_Study 3_ = 0.70). We therefore drop this item in the measure’s computation. These three items load onto one factor and onto one component, in all three studies.

*Stereotypes.* The six items load onto one factor and onto one component, in all three studies.

*Threat.* The five items load onto one factor and onto one component, in all three studies.

*Common identity.* The three items load onto one factor and onto one component, in all three studies.

*Support for aggressive policies against Muslims.* The five items load onto two factors in Studies 1 and 3. However, for both these studies, the items load onto one component. Additionally, the items load onto one factor and onto one component in Study 2.

*Resource allocation.* The three items load onto one factor and onto one component, in all three studies.

**Dual identity groups pilot study: comparing dual identity perception of different minority groups in the United States**

The goal of the pilot study was to find the appropriate social context for Study 3. Americans’ perceptions of intergroup conflict with Muslims is a relatively recent phenomenon, crystallized by the terrorist events of September 11, 2001, and Muslim American currently constitute a very small percentage of the US population - 0.8% (Gallup, 2016). Although the specific context of intergroup relations with the Muslim world, which remains understudied topic in social psychology, is timely and important in its own right, it may represent a relatively unique instantiation of intergroup relations in the US context.

Moreover, the context of the Muslim American gateway group can be seen as one in which the gateway group represents an overlap of social categories from different dimensions (i.e., religious and national). On the other hand, the impact of gateway group identification in a "classic" dual identity context, in which the gateway group represents an overlap of social categories from the same dimension, still requires examination. Furthermore, according to the findings from Study 2, in the base line control condition participants associated the Muslim American gateway group more with their outgroup\Muslim identity (*M*=81.21, *SD*=24.70) than with their ingroup\American identity (*M*=65.97, *SD*=32.84, *t*(60)=-3.70, *p*<0.001). Therefore, it is not clear that the same effect will take place with a gateway group that is identified more with the ingroup than with the outgroup prior to the manipulation.

Accordingly, the purpose of the pilot study was to assess and compare how Americans perceive Muslim Americans and seven other dual identity groups with an American national identity component and another national identity component. This assessment would be on two relevant identity dimensions: how strongly members of the group are identified (a) as American and (b) with the other component of their dual identity. The eight groups considered were (a) Muslim Americans, (b) Chinese Americans, (c) Hispanic Americans, (d) Mexican Americans, (e) African Americans, (f) Irish Americans, (g) Italian Americans, and (h) Russian Americans. We chose these groups because they all represented the more common form of dual identity (i.e. two identities on the same dimension), and they provided variance in terms of group size, race, and status in the US. The results of the pilot study can thus be used to determine a dual identity group for Study 3 that substantially differs in its perceived identity pattern from Muslim Americans to test the robustness of the findings from Study 2.

**Method**

**Participants.** Due to the fact that the participants recruited for the pilot study would also serve as the sample for Study 3 for the within participant analysis, we used G*Power software (Faul et al., 2009) to determine the target sample size necessary for the analysis of the pilot study and Study 3 combined. Based on the results of Study 2, we aimed to obtain 0.90 power for the detection of a small-medium effect (f^2^ = 0.05) at the standard .05 alpha error probability. The target sample size necessary was 373 participants. In anticipation of attrition of approximately 25% of participants between the pilot Study and Study 3, the need to remove participants who failed the comprehension check, we collected data from 517 participants (274 men, 238 women, 5 other*; M_age_*=36.63 *years*, *SD*=11.32) in total. Participants were recruited via Mturk after agreeing to fill out a survey in exchange for a compensation of $0.50. This equates to an hourly rate of $7.50 given the anticipated time-to-completion of 4 minutes.

**Procedure and measures.** Participants in the pilot study were presented with 8 different minority groups in the United States, Muslim Americans (for the sake of replication), and 7 groups that represent dual identities from the same dimension: Chinese Americans, Hispanic Americans, Mexican Americans, African Americans, Irish Americans, Italian Americans, and Russian Americans. The groups were presented in randomized order, and for each group participants were asked to rate the extent to which they perceive the identity of the gateway group as ingroup (American) on a scale of 1-100, and then to rate the extent to which they perceive them as outgroup on a scale of 1-100. After rating all the groups, participants provided demographic information.

**Results**

As in Studies 1 and 2, participants identified Muslim American more with the outgroup (*M*=78.94, *SD*=24.93) than with the ingroup (*M*=70.33, *SD*=31.71, *t*(516)=-4.97, *p*<0.001). However, all the other groups were identified more with the ingroup than with the outgroup:

***Table 3****. Ingroup and outgroup identification for dual identity minority groups* *in America*

| Group | Ingroup identity | | Outgroup identity | | *t* | *p* |
| --- | --- | --- | --- | --- | --- | --- |
|  | Mean | SD | Mean | SD |  |  |
| Muslim | 70.33 | 31.71 | 78.94 | 24.93 | -4.97 | .000 |
| Chinese | 77.14 | 25.61 | 68.90 | 28.55 | 5.11 | .000 |
| Hispanic | 77.04 | 25.98 | 72.59 | 27.87 | 2.90 | .004 |
| Russian | 75.13 | 26.48 | 67.92 | 29.19 | 4.09 | .000 |
| Irish | 83.45 | 21.30 | 60.14 | 33.04 | 13.57 | .000 |
| African | 85.29 | 21.77 | 54.61 | 35.27 | 16.10 | .000 |
| Mexican | 75.27 | 27.47 | 69.81 | 28.83 | 3.12 | .002 |
| Italian | 83.46 | 20.58 | 61.87 | 32.22 | 13.38 | .000 |

**Discussion**

Other than Muslim Americans, all other groups presented in the pilot study were identified by participants with the ingroup identity more than with the outgroup identity. Thus, all of the groups were eligible candidates for the next study i.e. Study 3. We chose to focus on Mexican Americans as a potential gateway group between the United States and Mexico for two main reasons. First, due to the political discourse at the time of the study (March 2019) the relationship between the United States and Mexico was the most salient and tense of all potential intergroup relations. Second, despite the demographic significance of this population in the United States, it remains largely understudied (Straka, 2019).
